# Chloroplast Signaling Gates Thermotolerance in *Arabidopsis*

**DOI:** 10.1016/j.celrep.2018.01.054

**Published:** 2018-02-13

**Authors:** Patrick J. Dickinson, Manoj Kumar, Claudia Martinho, Seong Jeon Yoo, Hui Lan, George Artavanis, Varodom Charoensawan, Mark Aurel Schöttler, Ralph Bock, Katja E. Jaeger, Philip A. Wigge

**Affiliations:** 1 Sainsbury Laboratory, University of Cambridge, Cambridge, UK; 2 Department of Plant Molecular Biology, University of New Delhi, Delhi, India; 3 Department of Biochemistry, Faculty of Science, and Integrative Computational BioScience (ICBS) Center, Mahidol University, Bangkok 10400, Thailand; 4 Max-Planck-Institut für Molekulare Pflanzenphysiologie, Potsdam-Golm, Germany

**Keywords:** Arabidopsis, thermotolerance, HSP70, diurnal, chloroplast, plastoquinone, HSF, light

## Abstract

Temperature is a key environmental variable influencing plant growth and survival. Protection against high temperature stress in eukaryotes is coordinated by heat shock factors (HSFs), transcription factors that activate the expression of protective chaperones such as *HEAT SHOCK PROTEIN 70* (*HSP70*); however, the pathway by which temperature is sensed and integrated with other environmental signals into adaptive responses is not well understood. Plants are exposed to considerable diurnal variation in temperature, and we have found that there is diurnal variation in thermotolerance in *Arabidopsis thaliana*, with maximal thermotolerance coinciding with higher *HSP70* expression during the day. In a forward genetic screen, we identified a key role for the chloroplast in controlling this response, suggesting that light-induced chloroplast signaling plays a key role. Consistent with this, we are able to globally activate binding of HSFA1a to its targets by altering redox status *in planta* independently of a heat shock.

## Highlights

• There is a diurnal pattern of basal thermotolerance in *Arabidopsis*
• Thermotolerance correlates with diurnal expression patterns of heat-associated genes
• Chloroplast mutants have greater heat shock gene expression and thermotolerance
• A chloroplast generated light signal gates HSFA1 and heat shock gene expression

## Graphical Abstract

**Figure:**
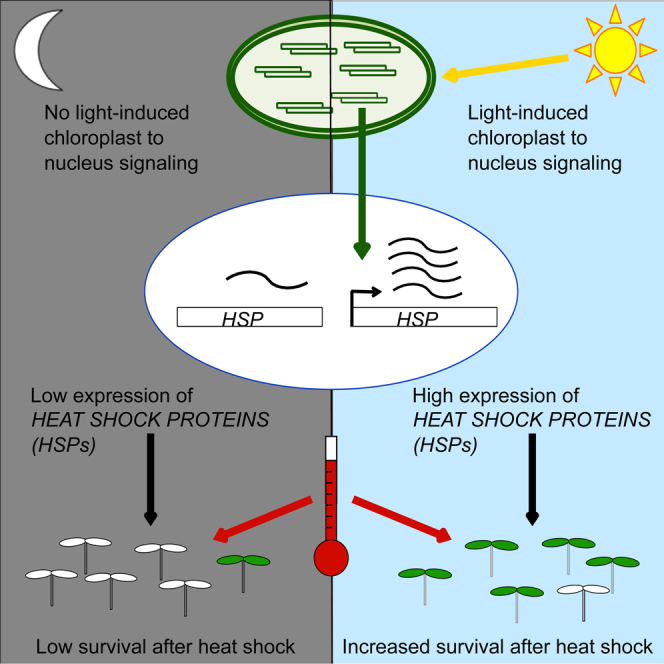

## Teaser

Plants are most resilient to heat stress during the day, a response controlled by HSFA1 transcription factors activating heat shock genes. Dickinson et al. find that perturbations of chloroplast electron transport affect heat shock gene expression. They show that HSFA1 activity is gated by a light-dependent chloroplast signal.

## Introduction

Temperature has a major role in plant growth and survival and therefore affects crop productivity. For example, wheat yields are predicted to decrease by ∼6% for every 1°C rise in global temperature (Asseng et al., 2014). High temperatures induce the expression of protective chaperones and modulate growth responses. Key players in the heat protection response are transcription factors of the HEAT SHOCK FACTOR A1 (HSFA1) family, which trigger the depletion of repressive H2A.Z-nucleosomes and target gene expression (Cortijo et al., 2017, Kumar and Wigge, 2010, Liu et al., 2011), however, the pathways that activate the HSFA1 class TFs and how these perceive temperature and integrate it with other environmental signals are not fully understood. Plants are exposed to considerable diurnal temperature variation and have evolved pathways to anticipate likely future conditions. For example, the cold response pathway is gated by the circadian clock, enabling the degree of responsiveness to be controlled in the context of the environment (Dodd et al., 2006, Lee and Thomashow, 2012) and genes promoting elongation growth and flowering in response to warm, non-stressful, temperature are induced during the night via thermosensory phytochromes (Jung et al., 2016, Legris et al., 2016). A diurnal pattern of thermotolerance has been reported in numerous plant species, including cereals (Laude, 1939), spinach (Li and Guy, 2001), plants using crassulacean acid metabolism (CAM) (Kappen and Lösch, 1984), and black spruce (Colombo et al., 1995). In this study, we find a diurnal pattern of thermotolerance in *Arabidopsis,* which correlates with the expression of *HSP70* over a diurnal cycle. A forward genetic screen with an *HSP70*-*LUCIFERASE* reporter line revealed a central role for light-induced chloroplast signaling in gating the level of response to high temperature that accounts for diurnal variation in thermotolerance.

## Results and Discussion

### Thermotolerance Varies Diurnally and Correlates with the Expression of *HSPs*

**Figure 1:**
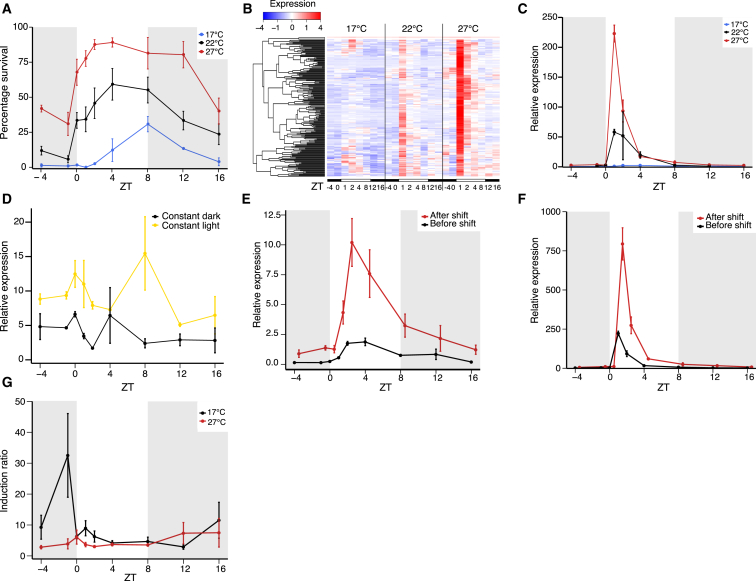
A Diurnal Pattern of Basal Thermotolerance Correlates with a Diurnal Pattern of Heat-Associated Gene Expression (A) Basal thermotolerance of WT seedlings over a diurnal time course. Error bars are ±SEM (n = 3), where 81 seedlings were scored per time point and temperature for three independent experiments. (B) Heatmap of the expression of genes in cluster 13-1-1. Transcriptomes shown from plants grown in short days (8 hr light, 16 hr dark) at constant 17°C, 22°C, or 27°C. Black bars below heatmap indicate samples taken in the dark (ZT−4, ZT0, ZT12, and ZT16) and white bars indicate samples taken in the light (ZT1, ZT2, ZT4, and ZT8). In heat maps, low to high expression (*Z* scores) is shown as blue to red. (C–F) Expression of *HSP70* in WT seedlings assayed by qRT-PCR. (C) Plants grown at 17°C, 22°C, and 27°C. Error bars are ± the range of two measurements (n = 2) or SEM (n = 3 to 4). (D) Seedlings entrained in short days at 27°C and shifted to constant light or constant dark at ZT8 of the seventh day after germination. Plants were sampled starting at ZT−4 on the eighth day. Error bars are ± the range of two measurements. (E and F) Before and after a 30-min shift to 45°C in seedlings grown at 17°C (E) and 27°C (F). (G) Induction ratios (expression after shift/expression before shift) of *HSP70* expression after the temperature shifts in (F) and (G). Error bars are ±SEM (n = 3).

To investigate whether the time of day gates thermotolerance in *Arabidopsis*, plants were grown for 7 days at constant 17°C, 22°C, or 27°C and survival after a heat treatment applied at nine points over a 24-hr time course was measured. We found that *Arabidopsis* has a diurnal pattern of thermotolerance (Figures 1A and S1A), consistent with reports from other plant species (Colombo et al., 1995, Kappen and Lösch, 1984, Laude, 1939, Li and Guy, 2001). The lowest levels of survival occur shortly before dawn and greatest survival occurs during the light period. The growth temperature affects the levels and patterns of thermotolerance, with higher growth temperatures resulting in increased thermotolerance occurring earlier in the day compared to lower temperatures (Figure 1A).

To identify gene expression changes underlying the diurnal pattern of thermotolerance, we analyzed transcriptomes of plants grown at constant 17°C, 22°C, and 27°C over a 24-hr time course. Genes were clustered based on expression patterns and a prominent cluster (cluster 13-1-1) showing a temperature responsive morning peak of expression was identified (Figure 1B). This morning cluster is strongly enriched for genes associated with the response to heat, high light, and oxidative stress (Table S2), including a number of *HEAT SHOCK PROTEINS* (*HSPs*) required for thermotolerance, such as *HSP101* (Queitsch et al., 2000), consistent with this cluster contributing to the diurnal pattern of thermotolerance. The promoters of genes in the morning cluster show enrichment for HSF binding sites known as heat shock elements (HSE) (Schöffl et al., 1998) (Table S3), suggesting that the temperature responsiveness of this cluster is HSF-dependent. Because *HSP70* expression is an indicator of temperature perception status (Kumar and Wigge, 2010) and is representative of the morning cluster (Figures 1C and S1B), we further analyzed *HSP70* to understand how the expression of this cluster of genes is controlled.

To test whether the diurnal pattern of *HSP70* expression is controlled by the circadian clock, plants were entrained in short days at constant 27°C before shifting to constant light or constant dark. Compared to plants grown at constant 27°C in short days (Figure 1C) there was an absence of the morning peak of *HSP70* expression in both constant light and constant dark (Figure 1D) indicating that the diurnal expression pattern is not primarily controlled by the circadian clock, consistent with work performed in spinach (Li and Guy, 2001). The morning peak was present, albeit substantially lower, in a *CIRCADIAN CLOCK ASSOCIATED 1* (*CCA1*) overexpressing line (*CCA1:OX*) (Figure S1C), which has an arrhythmic clock (Wang and Tobin, 1998). The lower morning peak in *CCA1:OX* may be the result of an indirect effect of altered reactive oxygen species (ROS) signaling in *CCA1:OX* (Lai et al., 2012). Supporting the idea that light regulation is dominant over circadian regulation of *HSP70* expression, the morning peak of LUCIFERASE activity in a line containing an *HSP70-LUCIFERASE* (*HSP70-LUC*) reporter (Kumar and Wigge, 2010) occurs 1 hr after lights come on when the night is extended by up to 4 hr (Figure S1D). The morning peak of *HSP70* expression is lost in both constant light and constant dark (Figure 1D) suggesting that it is dependent on the perception of the dark to light transition. The diurnal pattern of *HSP70* expression was present in *cryptochrome* and *phytochrome* null mutants and plants with a constitutively active phytochrome *(cry1/2*, *phyABCDE*, and *phyYHB*) (Jung et al., 2016) (Figures S1C and S1E) indicating that the morning peak is independent of the main blue (cryptochrome) and red/far red (phytochrome) photoreceptors, although the involvement of other photoreceptors has not been ruled out.

Because there is a diurnal pattern of *HSP70* expression at constant temperature (Figures 1C and S1B), we investigated whether the time of day also affects the induction of *HSP70* by heat. *HSP70* expression was assayed in plants grown at 17°C or 27°C and shifted to 45°C for 30 min, replicating the conditions of the thermotolerance assays. Levels of *HSP70* expression after a shift had a similar pattern to *HSP70* expression at constant temperature (Figures 1E and 1F) and this correlated with the diurnal patterns of thermotolerance (Figure 1A). The induction of *HSP70* by heat was quantified as the expression after shift divided by the expression before shift. Although there was large variability in *HSP70* expression levels between time points and growth conditions, induction ratios were similar (Figure 1G). The induction of *HSP70* expression in plants grown at 17°C and shifted at Zeitgeber time (ZT)−1 was higher, but this likely reflects experimental noise as the expression levels before the shift at ZT−1 are so low they are hard to measure accurately. There were also no large differences between induction ratios of plants grown at constant 17°C and 27°C, despite large differences in expression levels (Figures 1E–1G). The data are consistent with a model where light gates the magnitude of the response to high temperature, with expression after a temperature shift being a multiple of the expression before a shift. The expression of *HSP70* is very low in the dark, and consequently its expression at higher temperature is also minimal. By comparison, in the light a higher baseline expression of *HSP70* coincides with a proportionately higher expression level following induction. In this way, light can be thought as “priming” the heat shock response.

### Nuclear Genes Encoding Chloroplast Proteins Are Necessary for Correct HSP70 Expression during the Day, and This Correlates with Thermotolerance

**Figure 2:**
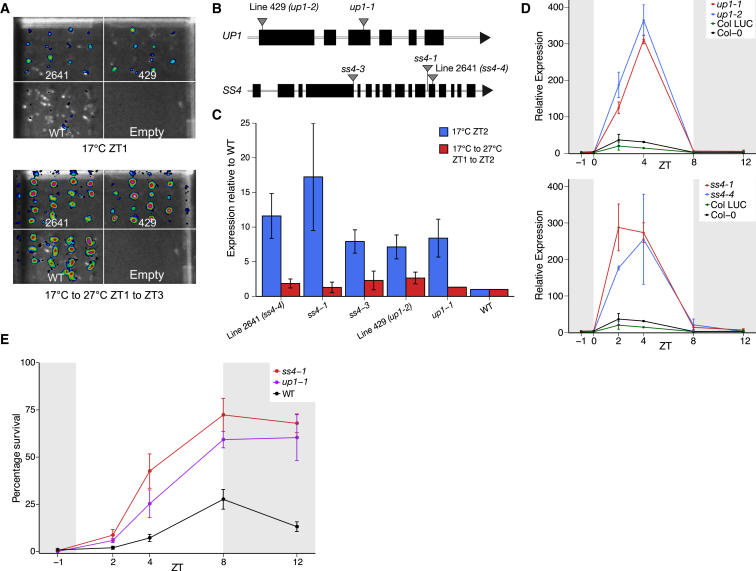
Mutations in Genes Encoding Chloroplast-Localized Proteins Cause Increased *HSP70* Expression and Increased Thermotolerance (A) False color image of LUCIFERASE activity of seedlings imaged at 17°C ZT1 and after a shift from 17°C to 27°C from ZT1 to ZT3. Two mutant lines, 2641 and 429, and WT (col-0 containing *pHSP70::LUC*), are shown from the same plate. (B) Schematics of *UP1* and *SS4* with the position of mutations shown. Line 429 (*up1-2*) has a C to T substitution on chromosome (chr) V at locus 2763941 mutating Gln10 to a stop codon. Line 2641 (*ss4-4*) has a C to T substitution on chr IV at locus 10086122 mutating Gln872 to a stop codon. (C and D) *HSP70* expression assayed by qRT-PCR. (C) Mutants at 17°C ZT2 and after a shift from 17°C to 27°C from ZT1 to ZT2. Expression shown relative to WT sampled from the same plate. Error bars are ± the range of two measurements (n = 2) or SEM (n = 3 to 4). (D) *up1* and *ss4* mutants over a time course at 17°C in short days sampled at ZT−1, ZT0, ZT2, ZT4, ZT8, and ZT12. Error bars are ± the range of two measurements (n = 2) or SEM (n = 3). (E) Thermotolerance of *up1-1*, *ss4-1*, and WT with plants grown at 17°C and treated at ZT−1, ZT2, ZT4, ZT8, and ZT12. Error bars are ±SEM (n = 3).

To investigate how the diurnal pattern of thermotolerance might be controlled, we screened for mutants with altered *HSP70* expression in the morning using the *HSP70-LUC* reporter line. Two mutant lines, 429 and 2641, were identified as having increased LUC activity in the morning at 17°C compared to wild-type (WT) (Figure 2A). The causal mutation in line 429 was mapped to a stop codon in a gene encoding an unknown protein found to be localized in the thylakoid membrane (*AT5G08540*) (Reiland et al., 2011, Simm et al., 2013) and the causal mutation in line 2641 was mapped to a stop codon in *STARCH SYNTHASE 4* (*SS4*) (Figure 2B; Supplemental Experimental Procedures). A T-DNA insertion in the unknown protein (*up1-1*) shows increased *HSP70* expression compared to WT, similar to line 429, and two independent *ss4* T-DNA mutants (*ss4-1* [Roldán et al., 2007] and *ss4-3* [Crumpton-Taylor et al., 2013]) show increased *HSP70* expression compared to WT, similar to line 2641 (Figure 2C). Complementation crosses confirmed that the *HSP70-LUC* mis-expression phenotypes in lines 429 and 2641 were caused by loss of UP1 and SS4 function respectively as F1 plants of crosses between the EMS and T-DNA mutants fail to rescue the LUC activity phenotypes of the EMS lines, whereas crosses to WT do rescue (Figures S2A and S2B).

The *up1* and *ss4* mutants have increased *HSP70* expression at 17°C ZT2 (Figure 2C). To investigate whether increased *HSP70* expression in these mutants is specific to a particular time of day, *HSP70* expression was assayed from ZT−1 to ZT12. This time course showed that the *up1* and *ss4* mutants have greatly increased *HSP70* expression specifically during the day (Figure 2D). In WT plants, the diurnal pattern of *HSP70* expression correlates with basal thermotolerance (Figures 1A and 1C). Similarly, increased *HSP70* expression during the day in *up1* and *ss4* (Figure 2D) correlates with increased thermotolerance in these mutants (Figures 2E and S2C).

The diurnal pattern of *HSP70* expression is light-dependent but broadly circadian clock and photoreceptor independent (Figures 1D and S1C–S1E) necessitating the involvement of another pathway to generate the morning peak of *HSP70* expression and therefore increase thermotolerance in response to light. As well as being the site of photosynthesis, the chloroplast is an environmental sensor and can transmit signals to effect nuclear gene expression through retrograde signaling (Chan et al., 2016), including expression of an *HSP70* gene in *Chlamydomonas* (Kropat et al., 1997). Both *UP1* and *SS4* encode chloroplast-localized proteins suggesting that the chloroplast is involved in generating the morning peak of *HSP70* expression. This is supported by the observation that the morning peak of *HSP70* expression is absent in light-exposed roots (Figure S2D) and that mutations in other genes encoding chloroplast-localized proteins, including *maltose exporter1* (*mex1*) (Niittylä et al., 2004) and *clpc1* (Sjögren et al., 2014) were identified in the screen for *HSP70-LUC* mis-expression and these mutants also have increased *HSP70* expression specifically during the day (Figure S2E).

### Increased Expression of Heat-Associated Genes in *ss4* Is Associated with the Light Reactions of Photosynthesis

**Figure 3:**
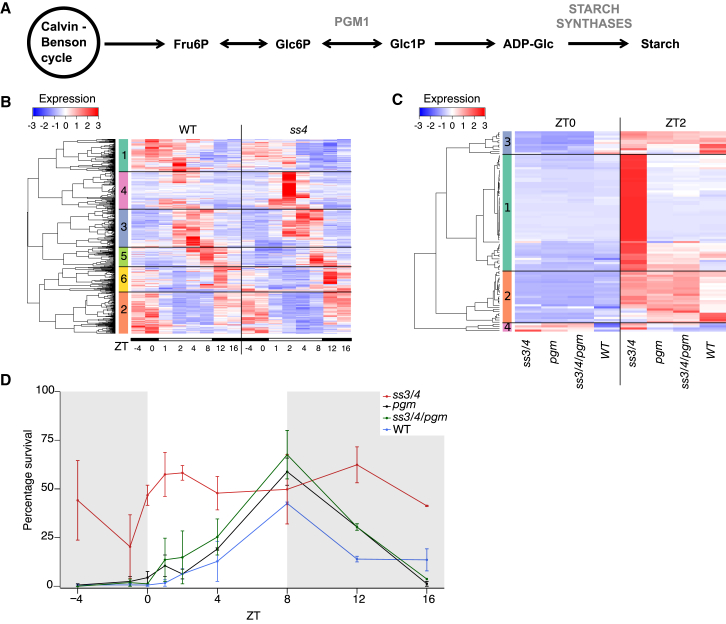
Increased Expression of Heat-Associated Genes in *ss4* Is Caused by Alterations to the Light Reactions of Photosynthesis (A) Simplified schematic of the starch synthesis pathway. (B) Heatmap of the expression of genes differentially expressed between *ss4-1* and WT at any sampled time-point. Transcriptomes are shown from plants grown in short days at 17°C. Black bars underneath heatmap show samples taken in the dark (ZT−4, ZT0, ZT12, and ZT16) and white bars show samples taken in the light (ZT1, ZT2, ZT4, and ZT8). Replicate shown in Figure S3A. (C) Heatmap of expression of cluster 4 from (B) in *ss3/4*, *pgm*, *ss3/4/pgm*, and WT (col-0) at 17°C ZT0 and ZT2. Replicate shown in Figure S3C. (B and C) Clusters shown on the left of heat maps and low to high expression (*Z* scores) is shown as blue to red. (D) Basal thermotolerance of *ss3/4*, *pgm*, *ss3/4/pgm*, and WT (col-0) grown at 17°C in short days and treated at ZT−4, ZT−1, ZT0, ZT1, ZT2, ZT4, ZT8, ZT12, and ZT16. Error bars are the range of two measurements.

As the function of *SS4* is well-characterized (Roldán et al., 2007, Crumpton-Taylor et al., 2013, Ragel et al., 2013), we used it to further characterize how chloroplast signaling affects diurnal patterns of heat-associated gene expression and thermotolerance. SS4 is a soluble starch synthase that catalyzes the addition of glucose from ADP-glucose to amylopectin chains (Streb and Zeeman, 2012) (Figure 3A). There are four soluble starch synthases in *Arabidopsis* and *ss4* is the only single mutant with a clear phenotype, likely because of its involvement in initiating starch granule synthesis (Crumpton-Taylor et al., 2013).

Expression of *HSP70* in *ss4* at 17°C shows the strongest difference to WT in the morning (Figure 2D), suggesting that the *ss4* mutant may affect the regulation of the morning cluster of genes (Figure 1B). To confirm this, we analyzed the *ss4* transcriptome over a diurnal time course at 17°C. Differentially expressed genes between *ss4* and WT were defined using DESeq2 (Love et al., 2014) and clustered based on expression patterns (Figures 3B and S3A). A cluster of genes (cluster 4) showing increased expression in *ss4* in the morning was strongly enriched for heat-associated genes (Table S2). This cluster overlapped with the cluster of morning peak genes (Figure S3B), and HSEs were enriched in the promoters of genes in this cluster (Table S3).

Defective starch synthesis leads to over-accumulation of ADP-glucose in *ss4* and *ss3/4* mutants (Ragel et al., 2013). This disrupts the photosynthetic electron transport (PET) chain as the over-accumulation of ADP-glucose limits the availability of ADP for the chloroplast ATP-synthase. This substrate-limitation results in decreased proton eflux from the thylakoid lumen through ATP synthase and thus an over-acidified lumen (Sharkey and Vanderveer, 1989, Takizawa et al., 2008). As a consequence, plastoquinol re-oxidation at the cytochrome b6f complex is slowed down (“photosynthetic control”) and non-photochemical quenching of excitation energy in the PSII antenna bed is strongly induced, something that has been suggested to initiate retrograde signaling cascades (Rott et al., 2011).

The *ss4* mutation affects both the carbon and light reactions of photosynthesis (Ragel et al., 2013) and causes increased expression of heat-associated genes in response to light (Figure 3B; Table S2). To determine whether the overexpression of heat-associated genes in *ss4* is associated with the carbon or light reactions of photosynthesis, we assessed the expression of cluster 4 (Figure 3B) in the transcriptomes of *ss3/4*, *plastidal phosphoglucomutase* (*pgm*), *ss3/4/pgm*, and WT at ZT0 and ZT2 at 17°C. Four clusters were defined and the genes in the largest cluster (cluster 4-1) were strongly overexpressed in *ss3/4* compared to WT in the morning (ZT2). This overexpression was absent in *pgm* and *ss3/4/pgm* (Figures 3C and S3C). Cluster 4-1 is strongly enriched for heat-associated genes and for genes with HSEs in their promoters (Table S3). *pgm* has very low levels of starch and increased levels of soluble sugars compared to WT (Ragel et al., 2013, Streb et al., 2009), therefore, effects on the carbon reactions do not cause increased expression of heat-associated genes as these genes are expressed similarly to WT in *pgm* (Figures 3C and S3C). Phenotypes associated with the light reactions such as photochemical efficiency, quantum yield of photosystem two, and an over-reduction of the plastoquinone (PQ) pool, are strongly affected in *ss4* and *ss3/4* compared to *pgm* (Ragel et al., 2013) suggesting that the mis-expression of cluster 4-1 in *ss4* and *ss3/4* is caused by alterations to the light reactions. Interestingly, the overexpression of cluster 4-1 is rescued in an *ss3/4/pgm* mutant, in which the over-accumulation of ADP-glucose in *ss3/4* is suppressed (Ragel et al., 2013), suggesting that the over-accumulation of ADP-glucose and the subsequent effects on the light reactions are the cause of the overexpression of heat-associated genes in *ss4* and *ss3/4.* The expression patterns of heat-associated genes in *ss3/4*, *pgm*, and *ss3/4/pgm* correlate with thermotolerance (Figures 3D and S3D) consistent with the central role of the light-induced morning cluster in determining thermotolerance.

### The Morning Peak of Heat-Associated Gene Expression Is Associated with a Reduction of the PQ Pool

**Figure 4:**
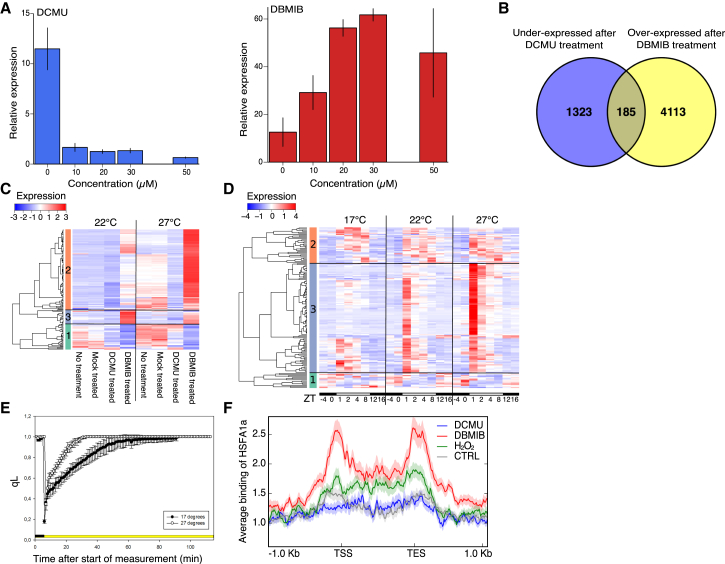
The Redox State of the Plastoquinone Pool Is Associated with the Expression of the Morning Cluster of Heat-Associated Genes (A) *HSP70* expression assayed by qRT-PCR after DCMU and DBMIB treatment of seedlings grown at 22°C in short days. Plants treated at ZT−1 and sampled at ZT1. Error bars are ±SEM (n = 3). (B) Overlaps of genes under-expressed (Log2 fold change <−0.5) after DCMU (30 μM) and overexpressed (Log2 fold change >0.5) after DBMIB (50 μM) treatments compared to a mock treatment at 22°C, treated at ZT−1 and sampled at ZT1. (C) Expression of cluster 13-1-1 (from Figure 1C) in transcriptomes of untreated, mock-treated, DCMU, and DBMIB-treated seedlings at 22°C and 27°C ZT1 after treatment at ZT−1. Replicate shown in Figure S3E. (D) Expression of the genes under-expressed after DCMU treatment and overexpressed after DBMIB treatment (overlap from B) in WT over a 24-hr time course (same as used for Figure 1C) at 17°C, 22°C, and 27°C. Black bars below heatmap indicate samples taken in the dark (ZT−4, ZT0, ZT12, and ZT16) and white bars indicate samples taken in the light (ZT1, ZT2, ZT4, and ZT8). (C and D) Clusters are shown on the left of heatmap and low to high expression (*Z* scores) is shown as blue to red. (E) qL over dawn at 17°C and 27°C. Error bars are ±SD (n = 4). (F) Average binding of HSFA1a to genes from the intersection of Figure 4B after treatment at ZT−1 with DCMU (30 μM), DBMIB (50 μM), H_2_O_2_ (5 mM), and a mock ethanol treatment. Plants sampled at ZT1. Shading represents SE. Replicate shown in Figure S3F.

Diurnal patterns of heat-associated gene expression and thermotolerance are affected by the light reactions of photosynthesis and the redox state of the PQ pool has been shown to affect the expression of heat-associated genes in response to excess light (Jung et al., 2013). Because of this and the observation that the PQ pool is more reduced in *ss4* than WT, and that this over-reduction is rescued in *ss3/4/pgm* (calculated using data from Ragel et al. [2013]; Supplemental Experimental Procedures), we investigated whether the redox state of the PQ pool affects *HSP70* expression. The redox state of the PQ pool can be modified experimentally by the application of 3-(3,4-dichlorophenyl)-1,1-dimethylurea (DCMU) and 2,5-dibromo-3-methyl-6-isopropyl-p-benzoquinone (DBMIB). DCMU prevents plastoquinol binding to Q_B_ and thus blocks electron transfer to the PQ pool, resulting in a more oxidized PQ pool (Pfalz et al., 2012). DBMIB prevents the PQ pool from transferring electrons to the cytochrome b_6_f (cyt-b_6_f) complex, resulting in a reduced PQ pool (Pfalz et al., 2012). We see that the morning peak of *HSP70* expression is abolished by DCMU, while it is increased by DBMIB, indicating that a reduced PQ pool is required for *HSP70* induction in the morning (Figure 4A).

As DCMU and DBMIB treatments oppositely affect *HSP70* expression, we examined which genes were affected in a similar way, as the expression of these genes may also be affected by the redox state of the PQ pool. At 22°C DBMIB treatment resulted in the overexpression of 4,298 genes and DCMU treatment resulted in the under-expression of 1,508 genes (Figure 4B). Despite the chemical treatments resulting in large changes to the transcriptome, only a small set of genes were both overexpressed after DBMIB and under-expressed after DCMU treatment (Figure 4B). This set of genes was strongly enriched for genes associated with responses to heat (Table S2) and for genes with HSEs in their promoters (Table S3) suggesting that the redox state of the PQ pool affects the morning peak of heat-associated gene expression. This is supported by the observation that the expression of the majority of genes in the morning cluster (Cluster 13-1-1 from Figure 1B) are increased by DBMIB treatment and decreased by DCMU treatment (Figure 4C and S3E). Similarly, the majority of genes that are oppositely affected by DCMU and DBMIB treatment have a morning peak of expression in untreated samples (Figure 4D).

As a reduced PQ pool is associated with the morning peak of heat-associated gene expression, we predicted that the PQ pool would be reduced by light in the morning. As expected the PQ pool was fully oxidized during the night, based on an indirect measure of the redox state of the PQ pool, qL (Kramer et al., 2004). With the onset of light qL decreased and then rose throughout the first hour of the day (Figure 4E), reflecting a reduction of the PQ pool by light and a subsequent re-oxidation when the Calvin-Benson cycle became light-activated. qL values decreased to a lower level at 17°C compared to 27°C and the amount of time until qL returned to one was slower at 17°C compared to 27°C, likely due to a slower induction of the Calvin-Benson cycle (Figure 4E). This indicates that the PQ pool becomes more reduced by light at lower temperatures; therefore, the level of reduction of the PQ pool does not appear to transmit temperature information in the ambient range. In *Chlamydomonas*, it has been proposed that light and temperature signals are integrated independently via Mg-protoporphyrin IX (MgProto) and HSFs, respectively (von Gromoff et al., 2006), however, the role of MgProto in retrograde signaling has been questioned (Moulin et al., 2008, Mochizuki et al., 2008). Because HSFs have previously been shown to respond to redox status (Jung et al., 2013, Miller and Mittler, 2006, Volkov et al., 2006), we investigated whether they might integrate both light and heat signals for morning cluster gene expression in *Arabidopsis*. The HSFA1 clade of HSFs have been shown to be essential for the early transcriptional response to heat (Liu et al., 2011), so we investigated the binding of HSFA1a to genes overexpressed after DBMIB treatment and decreased after DCMU treatment (intersection of Figure 4B) by chromatin immunoprecipitation sequencing (ChIP-seq). Interestingly, we observe a strong induction of HSFA1a binding to these genes in response to DBMIB at 22°C, while DCMU prevents the binding of HSFA1a to these genes (Figures 4F and S3F). Taken together, these results are consistent with a model where HSFA1a is able to integrate both the light and temperature signals, with the light signal being either PQ redox state or associated H_2_O_2_. Direct H_2_O_2_ transfer from the chloroplast to nucleus has been shown to be involved in high light signaling and was repressed by DCMU treatment (Exposito-Rodriguez et al., 2017). As H_2_O_2_ can activate HSFs (Volkov et al., 2006) and cause increased *HSP70* expression (Figure S3G), a model where H_2_O_2_ is produced in the chloroplast at the onset of light and transferred to the nucleus to activate the expression of heat-associated genes is an attractive model for generating diurnal patterns of heat-associated gene expression and thermotolerance.

### Conclusions

We find a diurnal pattern of thermotolerance in *Arabidopsis* that is a consequence of a gating effect of light-induced chloroplast signaling on the expression levels of heat-associated genes such as *HSP70*, accounting for a long-standing observation that plants have a differential ability to withstand heat stress in the day and night (Laude, 1939).

Under constant temperature conditions, the expression of morning peak genes such as *HSP70* is repressed into the afternoon (Figures 1B and 1C), likely because of repressive interactions of HSP70 and HSP90 with HSFA1s (Ohama et al., 2016) resulting in a negative feedback loop. The sharpness of the morning peak may also be influenced by growth chambers, with plants being subjected to a sudden change from darkness to full light, while plants in the field will experience a more gradual rise in light levels. Protective chaperones such as HSP70 have ATPase activity, and at high expression levels, can represent a significant energy demand. *Drosophila* cells overexpressing *HSP70* show greatly attenuated growth (Feder et al., 1992), and constitutive activation of heat-associated gene expression in *Arabidopsis* likewise greatly reduces growth (Ogawa et al., 2007). It may therefore be advantageous to limit heat-associated gene expression to the daytime, when temperature stress is most probable.

## Experimental Procedures

### Plant Material and Growth Conditions

Growth conditions were often specific to the experiment performed and these are described in the Supplemental Experimental Procedures.

For details of mutant lines used in this study see the Supplemental Experimental Procedures and Table S1.

### Thermotolerance Assays

A thermotolerance assay was developed based on a previously described protocol (Silva-Correia et al., 2014). After 7 days of growth, plates were floated in a water bath preheated to 45°C for 30 min. Survival was defined as the ability to produce new green leaves.

### Gene Expression Analysis

For qRT-PCR assays, RNA was extracted using a previously described phenol:chloroform extraction method (Box et al., 2011). Transcript levels were quantified using SybrGreen (Roche) and samples assayed with technical triplicates and on a LightCycler 480 (Roche).

### Identifying Candidate Mutants and Mapping Causal Genes

A previously described fusion between the promoter of *HSP70* and *LUCIFERASE* (*pHSP70::LUC*) (Kumar and Wigge, 2010) was used to screen EMS mutagenized Col-0 plants. LUC activity was imaged using a Photek HRPCS218 camera. Plates were imaged at 17°C at ZT1 and shifted to 27°C for 2 hr and imaged again at ZT3. Causal genes in were mapped by sequencing F2 plants displaying mutant phenotypes after outcrossing to *Landsberg erecta.*

### Transcriptomics

The MagMax RNA extraction kit (Ambion) was used to extract RNA from 20–25, 8-day-old seedlings and libraries prepared using the Illumina TruSeq stranded kit. Sequencing was performed on a NextSeq500 (Illumina) and an in-house RNA sequencing (RNA-seq) processing pipeline was used to process sequence data.

### ChIP-Seq

Chromatin was extracted from 1 g of cross-linked plant material, fragmented by sonication and ChIP was performed using anti-FLAG M2 magnetic beads (Sigma, M8823) coupled to a 1/1 mix of protein-A and protein-G Dynabeads (Life Technologies, 10001D and 10003D). ChIP-seq libraries were prepared using a TruSeq ChIP Library kit (Illumina) and sequenced on a NextSeq 500 (Illumina).

### Chlorophyll Fluorescence

The Maxi version of the Imaging-PAM M-series (Walz) was used to determine chlorophyll-a fluorescence parameters of intact plants. A custom-made version of the 3010-GWK1 gas exchange chamber (Walz) was used to control humidity and temperature.

## Data and Software Availability

The accession number for the raw and processed data from the RNA sequencing experiments reported in this paper is GEO: GSE96041.

## Supplemental Information

Document S1. Supplemental Experimental Procedures, Figures S1–S3, and Table S1

Table S2. Enriched Gene Ontology Processes, Related to Figures 1, 3, and 4 Two Gene Ontology (GO) enrichment tools, GOrilla and AgriGO, were used for enrichment analysis and all genes that passed the filters used when calculating Z-scores were used as background. The top five most enriched GO terms from each analysis are shown.

Table S3. Enriched Promoter Motifs, Related to Figures 1, 3, and 4 Promoter motif enrichment was performed using HOMER2. Sequences 1 kb upstream of the TSS were obtained from TAIR and these were used to search for known motifs. Sequences 1 kb upstream of all genes that passed the filters used when calculating Z-scores were used as background sequences. The top five most enriched motifs are shown.

Table S4. List of Genes Found in the Clusters Described in This Study, Related to Figures 1, 3, and 4

Document S2. Article plus Supplemental Information
