# Supplementary material for: Chloroplast Signaling Gates Thermotolerance in Arabidopsis
Source: Cell Rep. 2018 Feb 13;22(7):1657–65. doi: 10.1016/j.celrep.2018.01.054 (PMC5847188; doi:10.1016/j.celrep.2018.01.054)
Supplement: Document S1. Supplemental Experimental Procedures, Figures S1–S3, and Table S1 [file mmc1.pdf]

Cell Reports, Volume *22*

Supplemental Information

Chloroplast Signaling Gates Thermotolerance in *Arabidopsis*

Patrick J. Dickinson, Manoj Kumar, Claudia Martinho, Seong Jeon Yoo, Hui Lan, George Artavanis, Varodom Charoensawan, Mark Aurel Schöttler, Ralph Bock, Katja E. Jaeger, and Philip A. Wigge

Supplementary materials

Supplementary experimental procedures

Plant material and growth conditions

Arabidopsis thaliana WT plants were Columbia-0 (Col-0), Landsberg *erecta* (Ler) or *HSP70-LUC* where specified. The *HSP70-LUC* line has been reported previously (Kumar and Wigge, 2010) and a Ler *pHSP70::LUC* line was generated, by introgression of the *pHSP70::LUC* reporter from Col-0 *pHSP70::LUC.* T-DNA insertion mutants were obtained from the Nottingham Arabidopsis Stock Centre (NASC). Other mutants were have been reported previously: the *CCA1:OX* (Wang and Tobin, 1998) line was obtained from Dr Benoit Landrien (SLCU), *mex1-1* (Niittylä et al., 2004) seeds were obtained from Professor Sam Zeeman (ETH Zurich), and the *ss3/4*, *pgm* and *ss3/4/pgm* (Ragel et al., 2013) lines were obtained from Professor Ángel Mérida (University of Seville).

For general growth, *Arabidopsis* was grown in MTPS walk-in chambers (Conviron) at 22°C long days (16 hr light, 8 hr dark), under 170 µmol m^{-2} s^{-1} Photosynthetically Active Radiation (PAR) and 65% humidity. *Arabidopsis* was grown in Levington F2 compost in either PT24 trays or 7 cm^2 pots. Growth conditions were often specific to the experiment performed and these are described where relevant.

Thermotolerance assays

A thermotolerance assay was developed based on a previously described protocol (Silva-Correia et al., 2014). For each 10 cm^2 plate, 81 seeds were sown with even spacing on 35 ml ½ MS agar and stratified for 2-3 days at 4°C. Seeds were then germinated at 22°C for 24 hrs. After germination, seedlings were grown for seven days in short days (8 hrs light, 16 hrs dark) at 17°C, 22°C or 27°C. After seven days of growth, plates were floated on water in a water bath preheated to 45°C for 30 min. Plates were then put back to the conditions in which the plants were grown before treatment. If the time point when plates were treated was in the dark period, treatment was done with lights off in a dark room; if the heat treatment was done in the light period then treatment was done under ambient laboratory lights (ZT0 treated plants were treated in the light and ZT8 treated plants were treated in the dark (in short days)). Seven days after heat treatment, plants were imaged and the number of plants surviving scored. Survival was defined as the ability to produce new green leaves.

Gene expression analysis

The expression of *HSP70* was assayed by qRT-PCR in multiple experiments. These were broadly split into five categories; timecourse experiments, temperature shift experiments, mutant experiments, detached root and shoot experiments, and drug treatment experiments.

Timecourse experiments were performed at either 17°C, 22°C or 27°C in short days. Seeds were sown on 10 cm^2 plates containing 35 ml of ½ MS (pH 5.7) solid media and stratified for two to three days at 4°C in the dark. Seeds were germinated at 22°C for 24 hrs in short days then transferred to the required growth conditions. The WT short day (Figure 1A) timecourse experiment was grown in a Conviron PGC20 reach-in growth cabinet under 170 µmol m^2 s^{-1} white light. The constant light and dark (Figure 1D), *CCA1:OX* and *cry1/2* experiments (Figure S1C), and chloroplast mutant experiments (Figures 2D and S2E) were grown in a Sanyo-Panasonic MLR-352 growth cabinet under approximately 140 µmol m^2 s^{-1} white light. For timecourse experiments, Zeitgeber time (ZT) 0 indicated lights on and ZT0 samples were sampled in the dark. In short days ZT8 was the end of the day and ZT8 samples were sampled in the light. For constant light and constant dark experiments, plants were entrained to short days for seven days then shifted into constant light or constant dark growth chambers at ZT8 on the seventh day after germination.

For timecourse temperature shift experiments (Figures 1E and 1F) seedlings were grown as for timecourse experiments in Sanyo growth cabinets. On the eighth day after germination at ZT-4, -1, 0, 1, 2, 4, 8, 12 and 16, plates were shifted to 45°C for 30 min in a pre-heated water bath. Shifts at ZT-4, 8, 12 and 16 were performed in the dark, with the water bath in a room with no light, and shifts at 0, 1, 2 and 4 were performed in the light with the water bath under ambient laboratory lights. Samples were also taken just before plants were shifted.

For EMS and T-DNA mutant experiments (Figure 2C), plants were grown as for timecourse experiments in Sanyo cabinets at 17°C. Plates were shifted from a 17°C to 27°C at ZT1 and sampled after one hour at 27°C. A replicate plate was kept at 17°C and sampled at the same time (ZT2).

Root and shoot experiments (Figure S2D) were performed with plants grown vertically for three weeks in short days at 22°C on plate, in Sanyo cabinets. For the shoot and root samples, plants were cut to separate roots from shoots with a razor blade just before sampling.

To test the effects of chemicals on gene expression (Figure 4A), ten seedlings were grown in 12-well tissue culture test plates (Techno Plastic Products) in 500 µl of ½ MS liquid medium (pH 5.7) supplemented with 0.1% w/v glucose. Seedlings were stratified for two to three days at 4°C in the dark, germinated at 22°C for 24 hr and then grown in short days in Sanyo cabinets at either 17°C, 22°C or 27°C. 2-(3,4- dichlorophenyl)-1,1-dimethylurea (DCMU) (Sigma-Aldrich) and 2,5-dibromo-3-methyl-6-isopropyl-p-benzoquinone (DBMIB) (Sigma-Aldrich) were dissolved to 10 mM in 100% ethanol and these 10 mM stocks were diluted to 1 mM in 10% ethanol with sterile H_2O before use. H_2O_2 (Sigma-Aldrich) was diluted to a 50 mM working solution from a 30% w/v (9.8 M) stock in sterile H_2O. On the seventh day after germination plants were treated with 26.25 µl of the chemicals, diluted from 1 mM (DCMU and DBMIB) or 50 mM (H_2O_2) stocks to give the required final concentration in 523.25 µl. Plants were mock treated with either 26.25 µl of 10% ethanol or 26.25 µl of sterile H_2O depending on the chemical used. Plants were treated for the required length of time, depending on the experimental setup, then dried with tissue paper and flash frozen in liquid nitrogen.

For gene expression analysis by qRT-PCR, RNA was extracted from 20-25, seven day old seedlings using a previously described phenol:chloroform extraction method (Box et al., 2011). RNA was treated with RNase free recombinant DNase1 (Roche) to remove contaminating DNA and 1µg of this RNA was used for cDNA synthesis using the Transcriptor First Strand cDNA Synthesis Kit (Roche). Transcript levels were quantified by quantitative Reverse Transcription PCR (qRT-PCR) using SybrGreen (Roche) with 4 µl of cDNA (diluted 10x to a final concentration of 25 ng/µl) as template. Samples were assayed with technical triplicates and were run on a LightCycler 480 (Roche).

For timecourse experiments transcripts of *HSP70* were normalised to the geometric mean expression of *ASCORBATE PEROXIDASE 3 (APX3: AT4G35000)* and *ASPARTIC PROTEINASE A1 (APA1: AT1G11910)* as (Box et al., 2014). For temperature shift experiments transcripts of *HSP70* were normalised to *UBIQUITIN CONJUGATING ENZYME 21 (UBC21: AT5G25760)* (Czechowski et alk., 2005). For experiments where temperature shifts were performed at different times of day, transcripts of interest were normalised against both the geometric mean of *APA1* and *AP3* and against *UBC21*. The data reported are from normalisation against *UBC21* and results were very similar regardless of the reference genes used.

Identifying candidate mutants

A previously described fusion between the promoter of *HSP70 (pHSP70)* and *LUCIFERASE (pHSP70::LUC)* (Kumar and Wigge, 2010) was used to screen for *HSP70* expression in EMS mutagenized *Arabidopsis* Col-0 plants. Luciferase activity was imaged using a Photek HRPCS218 camera and a false colour map of the photon count was overlaid on a bright field image of the screened plate. Seedlings were sprayed with 1 mM D-luciferin free acid (SynChem) with 0.01% Triton X-100, so that all seedlings were covered, and then imaged. Plates were imaged at 17°C at ZT1 and shifted to 27°C for 2 hr and imaged again at ZT3.

Mapping causal genes

Homozygous M3 lines were crossed to Ler *HSP70-LUC* (Line 429) or Ler (line 2641). F2 lines from these crosses were then screened for segregating LUC activity to confirm the recessive nature of these mutations and to generate mapping populations.

Genomic DNA (gDNA) was extracted from a pool of 80-100 plants that showed the mutant phenotype in the F2 mapping populations. For isolation of nuclei and gDNA extraction, approximately 2 g of frozen leaf tissue was ground to a fine powder using pre-chilled pestles and mortars. The powder was re-suspended in 40 ml of ice-cold Honda buffer (25 mM Tris-HCl, pH 7.5, 0.44 M sucrose, 10 mM $MgCl_2$, 0.5% Triton X-100, 10 mM β-mercaptoethanol, 2 mM spermine), vortexed briefly and incubated on ice, with intermittent mixing, for 30 min. The resulting homogenate was filtered through a double layer of Miracloth into a 50 ml Falcon tube. The filtrate was then spun at 2,000 xg at 4°C for 15 min. The resulting pellet was re-suspended in 20 ml of Honda buffer and the suspension spun at 2000 xg for 15 min at 4°C. The resulting pellet was then re-suspended in 20 ml of Honda buffer without spermine and the suspension spun at 2000 xg for 15 min at 4°C. The resulting nuclei pellet was re-suspended in 500 µl of ice-cold TNE (10 mM TrisHCl, pH8.0, 100 mM NaCl, 1 mM EDTA) in four separate tubes. Next, 10 µl of RNaseA (Roche) (20 mg/ml) was added to each 1.5 ml tube (Eppendorf) and

samples were incubated at 65°C for 30 min. Samples were vortexed every 10 min through this heating step. Next 40 µl of proteinase K (AppliChem) (20 mg/ml) was added to each 1.5 ml tube and samples were incubated at 37°C for 60 min; samples were vortexed every 20 min during this heating step. After incubation, 500 µl of phenol:chloroform:isoamyl-alcohol (25:24:1, pH 7.5-8.5) was added and samples were vortexed and incubated on ice for 30 min with further vortexing every 5 min. Samples were then spun at 14,000 xg in a microcentrifuge for 10 min at 4°C. The supernatant was then transferred to new tubes, one volume of isopropanol and 1/10th volume of NaAC (3 M, pH 5.2) were added and samples incubated at -80°C for at least 40 min to precipitate DNA. Samples were then spun at 14,000 xg for 15 min at 4°C and the supernatant discarded. The resulting pellet was washed two times with 500 µl of ice-cold 70% ethanol and samples were spun at 14,000 xg for 5 min at 4°C between washes. The resulting pellet was then dried and re-suspended in 25 µl of TE buffer and the samples from the four separate tubes combined to a final volume of 100 µl. DNA concentration was measured using a Qubit fluorometer (Thermo Fisher scientific) using the high sensitivity, double stranded DNA protocol. DNA quality was assessed by agarose gel electrophoresis and good quality DNA was determined by the presence of a single, bright, high molecular weight band and the absence of a DNA smear.

Libraries for whole genome DNA sequencing were prepared using either the TruSeq DNA low throughput kit (Illumina) or the TruSeq PCR free low throughput kit (Illumina) as the manufacturer's instructions. DNA libraries were sequenced on a NextSeq2000 (Illumina) at the Beijing Genomics Institute (BGI), with 20 to 24 samples pooled per lane and pools sequenced with 100 bp paired end sequencing.

Bioinformatics analysis of the DNA sequencing was performed using CLC genomics (Qiagen). In this description, mapping population refers to pools of plants showing the mutant LUC phenotypes in F2 mapping populations.

Fastq reads from the sequenced mutants and from the parental Col-0 *pHSP70::LUC* line were mapped against the Col-0 (TAIR10) and the Ler reference genomes. Variants were then determined between Col-0 *pHSP70::LUC* and Ler and between the mapping population and Ler using the probabilistic variance detection function. Homozygous SNPs between Col-0 *pHSP70::LUC* and Ler and SNPs of any zygosity between the mapping population and Ler were used for mapping candidate regions. These SNPs were used for mapping candidate regions as they represent differences between the mapping population and Ler that were caused by the differences between the *pHSP70::LUC* parental line and Ler. These SNPs were then filtered for heterozygosity (SNP frequency between 35% and 65%) and for homozygosity (SNP frequency greater than 90%). Frequencies of heterozygous and homozygous SNPs across the genome were then visualised using the CLC visualisation tools, with 100 kb bins of SNP frequency plotted against chromosome position. SNP frequencies were then assessed visually for a region depleted in heterozygous SNPs and enriched for homozygous SNPs against Ler. This region was then classed as a candidate region.

To identify candidate mutations within the candidate region, reads from the mapping population were mapped against the Col-0 TAIR10 genome and variants between the mapping population and TAIR10 were determined using the probabilistic variance detection function. These variants were then filtered for homozygous (SNP frequency greater than 80%, to allow for some mis-scoring of F2 plants) EMS induced (GC to AT) SNPs that caused non-synonymous amino acid changes in the protein they encoded. SNPs that fulfilled these criteria and that were located in the candidate region were classed as candidate mutations.

Confirming causal genes

To identify causal mutations, T-DNA insertion lines in the genes harbouring candidate mutations were genotyped to identify lines homozygous for the required T-DNA insertion. Lines were genotyped using primers designed to bind 100-200 bp upstream (LP) and downstream (RP) of the annotated T-DNA insertion (Table S1). PCR was performed using LP and RP primers and a primer against the left border of the T-DNA insertion (primer LB1.3 (SALK)). Where available, T-DNA lines with insertions in exons of the gene of interest were obtained. These homozygous T-DNA lines were then screened for *HSP70* expression phenotypes by qRT-PCR and T-DNA mutants that had *HSP70* expression phenotypes similar to the corresponding EMS mutant lines were likely to be in the causal genes (As shown for lines 429 and 2641 in Figure 2C).

Transcriptomics

RNA-seq experiments were performed for WT at 17°C, 22°C and 27°C over a 24 h timecourse, *ss4* at 17°C over a 24 h timecourse, *ss3/4, pgm* and *ss3/4/pgm* at 17°C ZT0 and ZT2, and DCMU and DBMIB treated WT at 22°C, ZT1, all in short days. Two independent biological replicates were generated for WT and *ss4* at 17°C,

ss3/4, *pgm* and *ss3/4/pgm* at 17°C and 27°C, and DCMU and DBMIB treatment experiments, there is one biological replicate for WT at 22°C and 27°C. For RNA-seq experiments, all samples were grown in Conviron chambers and sampled as described for *HSP70* expression analysis.

The MagMax RNA extraction kit (Ambion) was used to extract RNA from 20 to 25, eight-day-old seedlings. Extractions were performed as the manufacturer's instructions. RNA was quantified using a Nanodrop 1000-spectrophotometer (Thermo Scientific) and RNA quality was assessed using the 2200 TapeStation system (Agilent) using an RNA1000 screentape (Agilent).

Libraries for RNA sequencing were prepared using either the Illumina TruSeq stranded low or high throughput library preparation kits (Illumina) with 1 µg of RNA as input. Libraries were prepared as the manufacturer's with one minor change; the number of PCR cycles for the DNA enrichment step was reduced from 15 to 13. Library quality was assessed using the Agilent 2200 TapeStation with the D1000 screentape kit.

Sequencing was performed on a NextSeq500 (Illumina) running a final pooled library concentration of 1.8 pM in 1.3 ml. Each pool contained 24 samples and was sequenced using high output, 75 bp, paired end sequencing, generating approximately 1,000,000,000 reads per run.

An in house RNA-seq processing pipeline was used to process the generated sequence data. First, read quality was assessed the quality of reads using FastQC (www.bioinformatics.babraham.ac.uk/projects/fastqc/). Adapter sequences were removed using Trimmomatic (version (v) 3.2) (Bolger et al., 2014), reads mapped to the TAIR10 genome using TopHat (v2.0.10) (Trapnell et al., 2010), duplicate reads removed using Picard's MarkDuplicates (Picard Tools, Broad Institute), transcript abundance estimates, RPKM (Reads Per Kilobase of transcript of transcript per Million mapped reads) and FPKM (Fragments Per Kilobase of transcript per Million mapped reads) were generated using Cufflinks (v2.2.1) (Trapnell et al., 2010) and TPM (Transcript Per Million) values were calculated using the formula described in (Wagner et al., 2012).

TPM values were used for subsequent analysis as they are the least biased estimate of transcript abundance (Wagner et al., 2012). For clustering, the data was filtered to remove both lowly and stably expressed genes using the following parameters. Genes where the sum of the TPMs from all samples in that experiment was less than then number of samples (mean TPM <1) were removed, as were genes where there were a high number of samples with a TPM of 0. The threshold number of 0 was subjective and varied depending on the number of samples in the experiment but roughly if a gene had more than 80% of samples with a TPM of 0 it was removed. A coefficient of variance (CV) (standard deviation of TPM of one gene between samples)/(average of TPM for the same gene between samples) was calculated for each gene and genes with a CV > 0.3 were kept. TPM values were then transformed to enable comparisons between samples by calculating Z-scores ((TPM for one gene in one sample) - (average of TPMs for the same gene between samples))/(standard deviation of TPMs for the same gene between samples) or by calculating Log2 ratios between samples of interest and a control sample (log2 (sample/control)).

The R package DEseq2 (Love et al., 2014) was used to determine differentially expressed genes with the raw read counts from two independent biological replicates used as inputs. For the *ss4* and WT 17°C timecourse, *ss4* samples were used as the samples of interest and WT at the same time points were used as the control samples. Differentially expressed genes were defined as having a log2 ratio of *ss4/*WT more than 0.5 or less than -0.5 and an adjusted p-value less than 0.05. The same cut offs were used for the DCMU and DBMIB experiments, with the mock (ethanol) controls used as the control samples. For the *ss3/4*, *pgm*, *ss3/4/pgm* experiment there were substantially more differentially expressed genes than the other experiments, therefore differentially expressed genes were defined as having a log2 ratio of sample/WT of more than 1 or less than -1, with an adjusted p-value less than 0.05.

Clustering of genes by expression was performed using the 'hclust' function on a 1-Pearson correlation matrix of either Z-scores or log2 ratios (sample/WT) of filtered TPM values and clustering was performed using the complete linkage method. The number of clusters was defined manually and gene expression was visualised using the R function heatmap.2.

Clustering for differentially expressed genes was performed using Z scores for differentially expressed genes. As there were two replicates for the *ss4* and WT at 17°C experiment, Z scores from only one of the replicates were used for clustering.

To determine the enrichments for biological functions in various samples, gene ontology analysis was performed using both the AgriGO tool (Du et al., 2010) and GOrilla (Eden et al., 2009) tools. Genes that passed the filters used when calculating Z-scores for the relevant transcriptomes were used as background.

Motif enrichment analysis was performed using the HOMER2 motif analysis tool (http://homer.salk.edu/homer/) (Heinz et al., 2010). Sequences 1 kb upstream of the transcription start site were analysed using the findmotifs.pl tool to search for enriched known motifs against a background of the 1 kb region upstream of genes that passed the filters used when calculating Z-scores for the relevant transcriptomes

Venn diagrams were produced to assess the numbers of overlapping genes between samples. Venn diagrams were produced using the Venny 2.1 tool (http://bioinfogp.cnb.csic.es/tools/venny/).

ChIP-seq

Plant material was immediately cross-linked when collected using 1% formaldehyde for 15 min under vacuum. Chromatin was extracted from 1 g of cross-linked material. Chromatin was fragmented by sonication using a Bioruptor (Diagenode) in lysis buffer (10 mM Tris-HCl [pH 8], 150 mM NaCl, 1 mM EDTA [pH 8], 0.1% deoxycholate, and 1X protease inhibitor cocktail). ChIP was performed in a buffer containing 20mM Tris-HCl (pH8), 150mM NaCl, 2mM EDTA, 1% triton X-100 and 1X protease inhibitor cocktail using anti-FLAG M2 magnetic beads (Sigma, M8823) coupled to a 1/1 mix of protein-A and protein-G Dynabeads (life technologies, 10001D and 10003D). ChIP-seq libraries were prepared using a TruSeq ChIP Library kit (Illumina) and sequenced on a NextSeq 500 (Illumina).

Chlorophyll fluorescence

The Maxi version of the Imaging-PAM M-series (Walz) was used to determine chlorophyll-a fluorescence parameters of intact plants. The Imaging-PAM was coupled to a custom-made version of the 3010-GWK1 gas exchange chamber (Walz), which can accommodate intact *Arabidopsis* plants in the rosette stage / prior to bolting and allows full control of humidity and temperature. Plants were dark-adapted for 30 min to ensure that all photosystem two (PSII) reaction centres were in the open state. Then, a weak pulse of measuring light (0.1 µmol quanta m^{-2} s^{-1}, 1 Hertz frequency) was applied to determine minimal fluorescence (F_o). Plants were then treated with a saturating light pulse (approximately 6000 µmol m^{-2} s^{-1}) to put all PSII reaction centres into a closed state and thus to determine maximal fluorescence (F_m). Then, plants were either kept in darkness, or the induction of photosynthesis was followed in actinic light, comparable to the growth light intensity. During photosynthetic induction, steady state fluorescence (F_s) was continuously measured, and maximum fluorescence levels in the light, F_m' , were determined every minute through the course of the experiment. The fraction of open PSII reaction centres according to the lake model of PSII reaction centre connectivity (qL) was calculated according to (Kramer et al., 2004).

Calculating PQ redox state

F_v/F_m and ϕPSII for Col-0, *ss4*, *ss3/4*, *pgm* and *ss3/4/pgm* were reported in (Ragel et al., 2013). The redox state of the PQ pool was calculated from these values using the formula qP= ϕPSII/(F_v/F_m) (Brooks and Niyogi, 2011). The calculated qP values using these measurements were: 0.488 for WT, 0.409 for *ss4*, 0.281 for *ss3/4*, 0.482 for *pgm* and 0.502 for *ss3/4/pgm*.

Supplemental references

Bolger, A.M., Lohse, M., and Usadel, B. (2014). Trimmomatic: A flexible trimmer for Illumina sequence data. Bioinformatics *30*, 2114–2120.

Box, M.S., Coustham, V., Dean, C., and Mylne, J.S. (2011). Protocol: A simple phenol-based method for 96-well extraction of high quality RNA from Arabidopsis. Plant Methods *7*, 7.

Box, M.S., Huang, B.E., Domijan, M., Jaeger, K.E., Khattak, A.K., Yoo, S.J., Sedivy, E.L., Jones, D.M., Hearn, T.J., Webb, A.A.R., *et al.* (2014). ELF3 Controls Thermoresponsive Growth in Arabidopsis. Curr. Biol. *25*, 194–199.

Brooks, M.D., and Niyogi, K.K. (2011). Chloroplast Research in Arabidopsis: Methods and Protocols. Volume II. Methods in Molecular Biology *775*, 299–310.

Czechowski, T., Stitt, M., Altmann, T., and Udvardi, M.K. (2005). Genome-Wide Identification and Testing of Superior Reference Genes for Transcript Normalization. Plant Physiol *139*, 5–17.

Du, Z., Zhou, X., Ling, Y., Zhang, Z., and Su, Z. (2010). agriGO: A GO analysis toolkit for the agricultural community. Nucleic Acids Res. *38*, 64–70.

Eden, E., Navon, R., Steinfeld, I., Lipson, D., and Yakhini, Z. (2009). GOrilla: a tool for discovery and visualization of enriched GO terms in ranked gene lists. BMC Bioinformatics *10*, 48.

Heinz, S., Benner, C., Spann, N., Bertolino, E., Lin, Y.C., Laslo, P., Cheng, J.X., Murre, C., Singh, H., and Glass, C.K. (2010). Simple Combinations of Lineage-Determining Transcription Factors Prime cis-Regulatory Elements Required for Macrophage and B Cell Identities. Mol. Cell *38*, 576–589.

Kramer, D.M., Johnson, G., Kiirats, O., and Edwards, G.E. (2004). New fluorescence parameters for the determination of Q(a) redox state and excitation energy fluxes. Photosynth. Res. 79: 209–218.

Kumar, S.V., and Wigge, P. A (2010). H2A.Z-containing nucleosomes mediate the thermosensory response in Arabidopsis. Cell *140*, 136–47.

Love, M.I., Huber, W., and Anders, S. (2014). Moderated estimation of fold change and dispersion for RNA-seq data with DESeq2. Genome Biol. *15*, 1–34.

Niittylä, T., Messerli, G., Trevisan, M., Chen, J., Smith, A.M., and Zeeman, S.C. (2004). A previously unknown maltose transporter essential for starch degradation in leaves. Science *303*, 87–89.

Ragel, P., Streb, S., Feil, R., Sahrawy, M., Annunziata, M.G., Lunn, J.E., Zeeman, S., and Mérida, Á. (2013). Loss of starch granule initiation has a deleterious effect on the growth of arabidopsis plants due to an accumulation of ADP-glucose. Plant Physiol. *163*, 75–85.

Silva-Correia, J., Freitas, S., Tavares, R.M., Lino-Neto, T., and Azevedo, H. (2014). Phenotypic analysis of the Arabidopsis heat stress response during germination and early seedling development. Plant Methods *10*, 7.

Trapnell, C., Williams, B. a, Pertea, G., Mortazavi, A., Kwan, G., van Baren, M.J., Salzberg, S.L., Wold, B.J., and Pachter, L. (2010). Transcript assembly and quantification by RNA-Seq reveals unannotated transcripts and isoform switching during cell differentiation. Nat. Biotechnol. *28*, 511–5.

Wagner, G.P., Kin, K., and Lynch, V.J. (2012). Measurement of mRNA abundance using RNA-seq data: RPKM measure is inconsistent among samples. Theory Biosci. *131*, 281–285.

Wang, Z.Y., and Tobin, E.M. (1998). Constitutive expression of the *CIRCADIAN CLOCK ASSOCIATED 1 (CCA1)* gene disrupts circadian rhythms and suppresses its own expression. Cell *93*, 1207–1217.

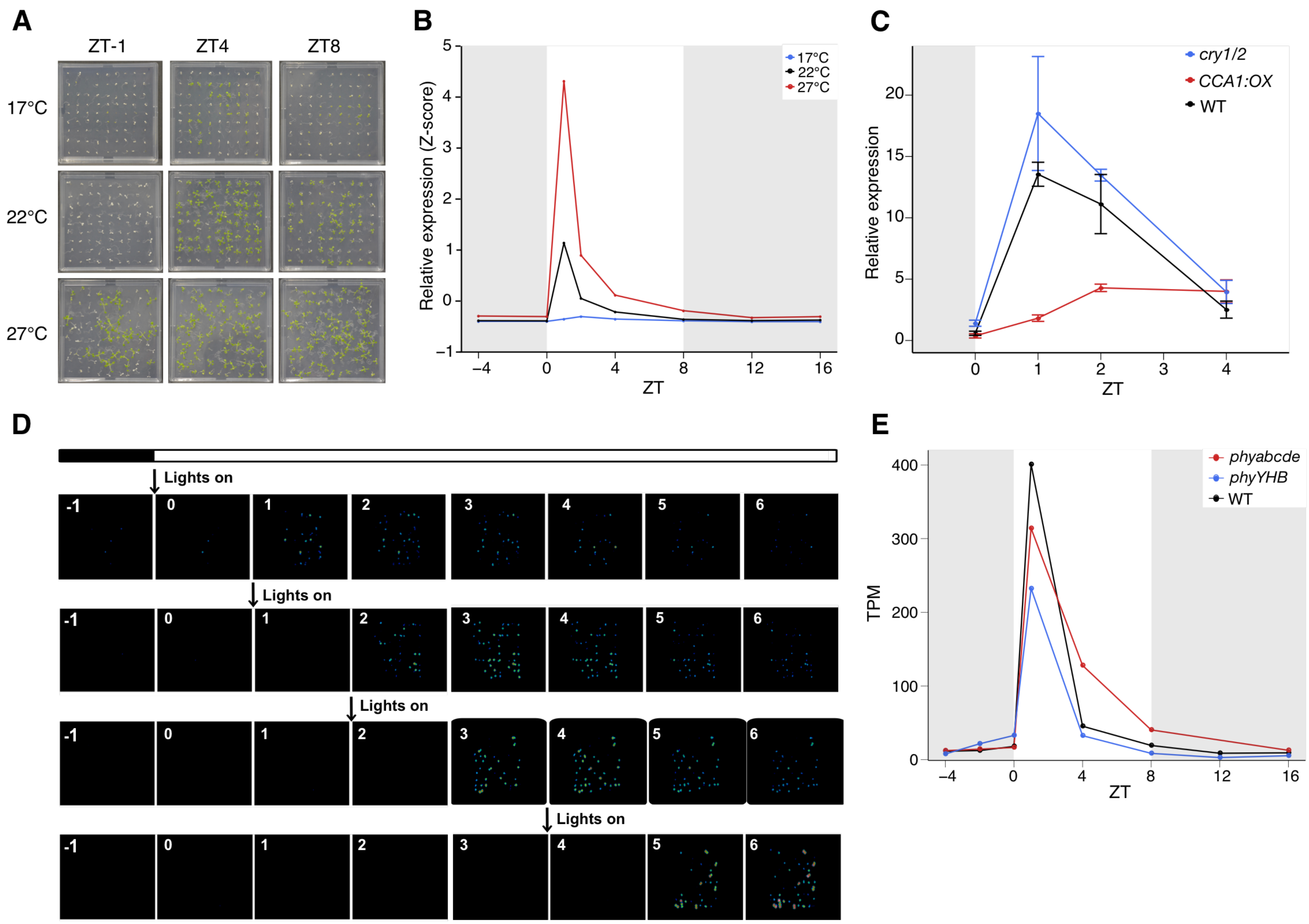
A
ZT-1
ZT4
ZT8
17°C
22°C
27°C
B
Relative expression (Z-score)
ZT
17°C
22°C
27°C
C
Relative expression
ZT
cry1/2
CCA1:OX
WT
D
Lights on
E
TPM
ZT
phyabcde
phyYHB
WT

Figure S1 (related to Figure 1)

A) Representative images of WT plants, eight days after heat treatment (45°C for 30 min). B) Expression of *HSP70* in the transcriptomes shown in Figure 1B. Expression shown as Z-scores. WT (Col-0) plants were grown at 17°C, 22°C and 27°C over a 24 h timecourse in short days, grey areas indicated lights off. C) *HSP70* expression assayed by qRT-PCR in WT (Col-0), *cry1/2* and *CCA1:OX* at 22°C ZT0, 1, 2, and 4 in short days. Error bars are + and – SEM (n=3). D) Night extension experiment. LUC activity of *pHSP70::LUC* plants grown in short days at 20°C and imaged at ZT-1, 0, 1, 2, 3, 4, 5, and 6. Each row is a different experiment with 0, 1, 2, and 4 h night extensions into the morning. The black and white bar above the images shows subjective night and day. E) Expression, shown as TPM, of *HSP70* in WT (Ler), *phyabcde* and *phyYHB* plants grown in short days at 22°C. Data from transcriptomes first published in (Jung et al., 2017).

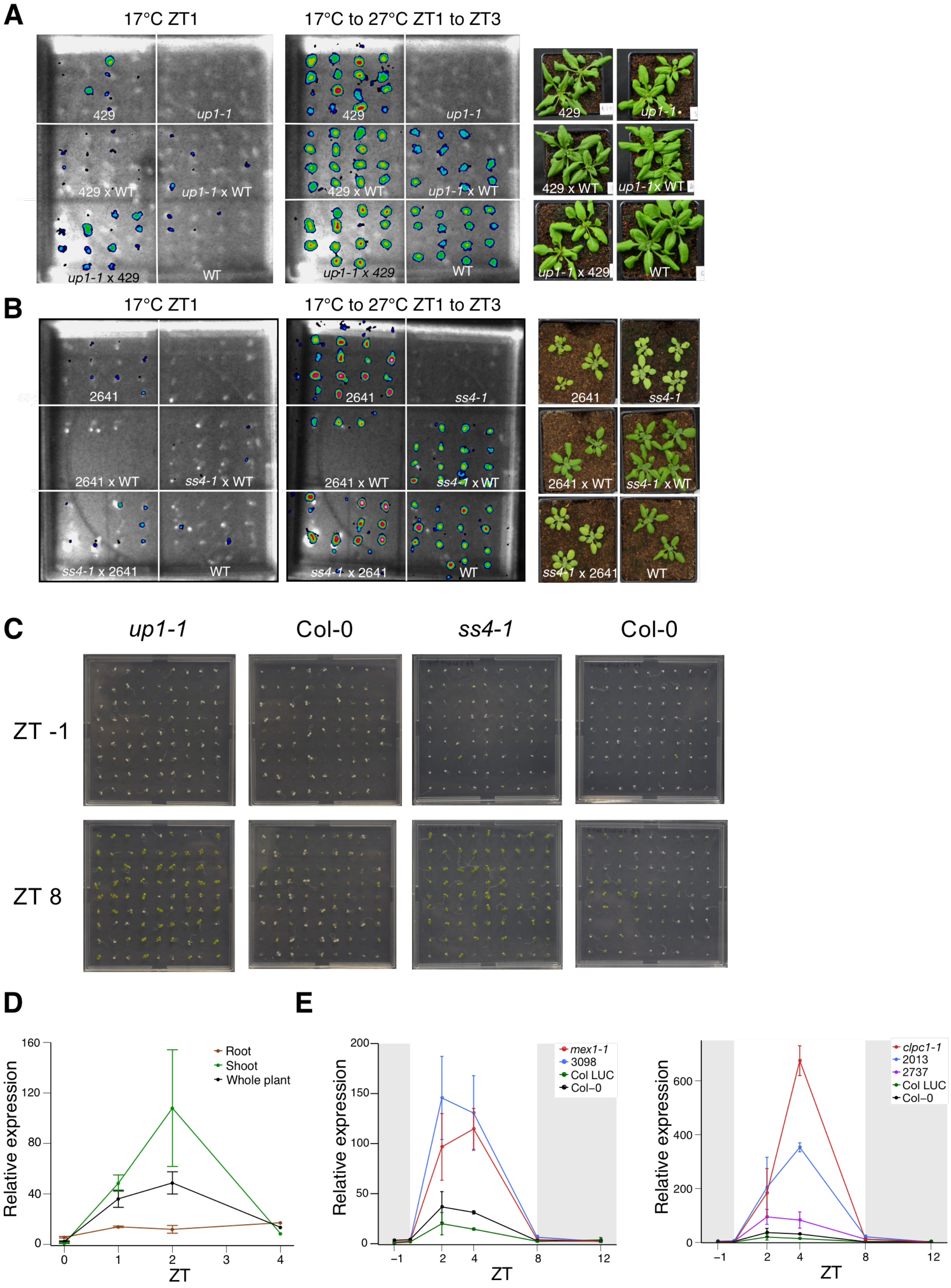

A
17°C ZT1
17°C to 27°C ZT1 to ZT3
429
up1-1
429 x WT
up1-1 x WT
up1-1 x 429
WT
B
17°C ZT1
17°C to 27°C ZT1 to ZT3
2641
ss4-1
2641 x WT
ss4-1 x WT
ss4-1 x 2641
WT
C
up1-1
Col-0
ss4-1
Col-0
ZT -1
ZT 8
D
Relative expression
ZT
Root
Shoot
Whole plant
E
Relative expression
ZT
mex1-1
3098
Col LUC
Col-0
clpc1-1
2013
2737
Col LUC
Col-0

Figure S2 (related to Figure 2)

A) LUC activity of F1 plants from complementation crosses between line 429 and *up1-1* at 17°C ZT1 and after a shift to 27°C for two hours from ZT1 to ZT3. Whole plant phenotypes are shown from the same crosses to the right of the LUC images. WT is Col *pHSP70::LUC.* B) As (A) for line 2641 and *ss4-1*. C) Representative images of *up1-1*, *ss4-1* and WT seedlings after heat treatment started at ZT-1 and ZT8 eight days after heat treatment. D) Expression of *HSP70*, assayed by qRT-PCR, in either roots, shoots, or whole WT plants grown at 22°C in short days and sampled at ZT0, 1, 2, and 4. Error bars are + and – SEM (n=3 for ZT0, 1, 2 and n=1 for ZT4). E) *HSP70* expression assayed by qRT-PCR in *mex1* (line 3098 and *mex1-1*) and *clpc1* (lines 2013 and 2737, and *clpc1*-1) mutants over a timecourse at 17°C in short days sampled at ZT-1, 0, 2, 4, 8 and 12. Grey areas indicate the dark period. Error bars are + and – the range of two measurements (n=2) or SEM (n=3).

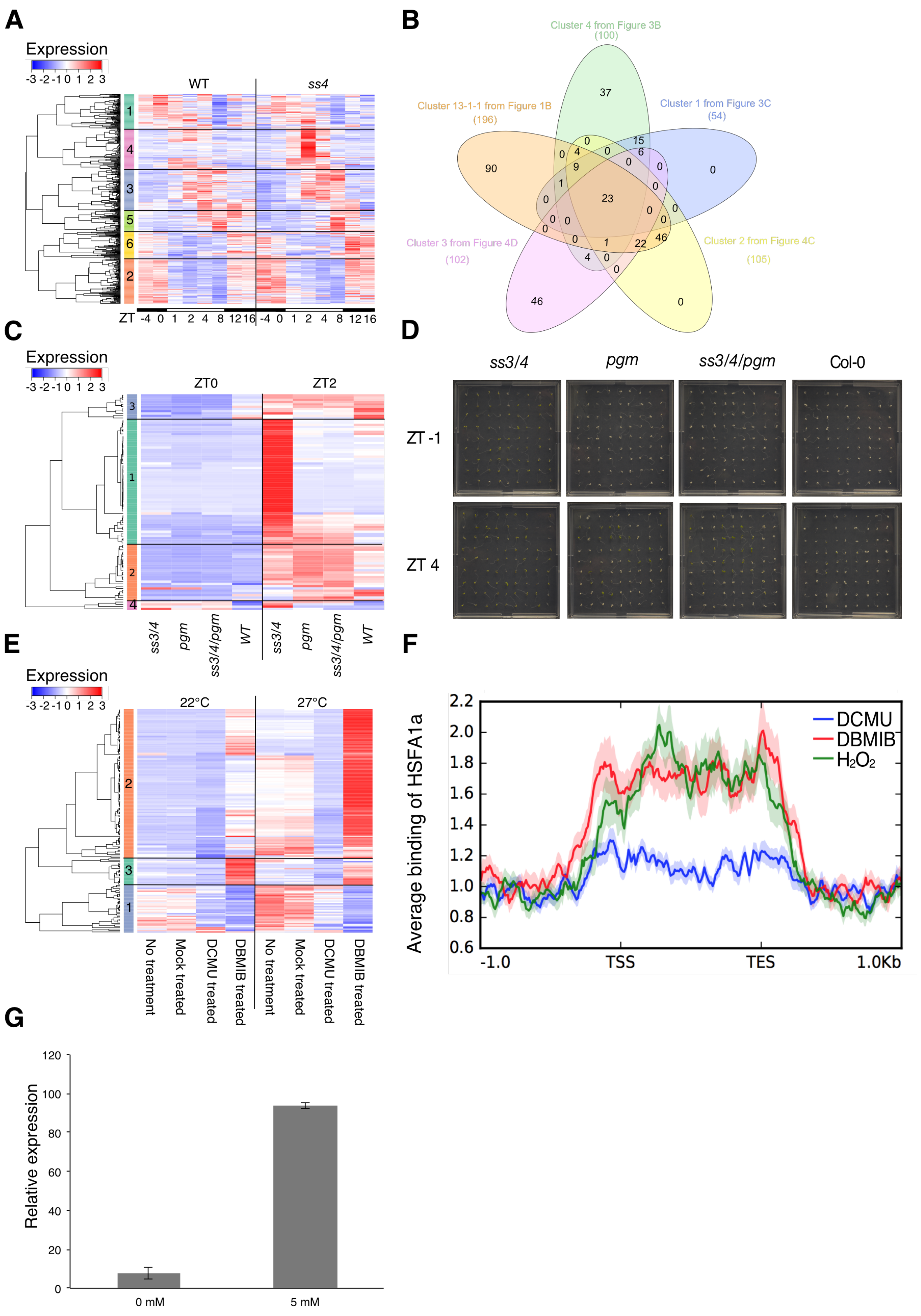
A
Expression
-3 -2 -1 0 1 2 3
WT
ss4
ZT -4 0 1 2 4 8 12 16 -4 0 1 2 4 8 12 16
B
Cluster 4 from Figure 3B (100)
Cluster 13-1-1 from Figure 1B (196)
Cluster 1 from Figure 3C (54)
Cluster 3 from Figure 4D (102)
Cluster 2 from Figure 4C (105)
C
Expression
ZT0
ZT2
ss3/4 pgm ss3/4/pgm WT ss3/4 pgm ss3/4/pgm WT
D
ss3/4
pgm
ss3/4/pgm
Col-0
ZT -1
ZT 4
E
Expression
22°C
27°C
No treatment
Mock treated
DCMU treated
DBMIB treated
F
DCMU
DBMIB
H2O2
Average binding of HSFA1a
-1.0
TSS
TES
1.0Kb
G
Relative expression
0 mM
5 mM

Figure S3 (related to Figures 3 and 4)

A) Clustering of differentially expressed genes between *ss4* and WT at 17°C over a 24 h timecourse (sampled at ZT-4, 0, 1, 2, 4, 8, 12, and 16) in a replicate of the transcriptomes shown in Figure 3B. Expression is shown as Z-scores and the clusters shown in Figure 3B were used for clustering. B) Overlaps between clusters of genes described throughout the manuscript. C) Clustering of cluster 6 from Figure 3B in a second replicate of transcriptomes of *ss3/4, pgm, ss3/4/pgm* and WT at 17°C ZT0 and 2. First replicate shown in Figure 3C and the clusters from Figure 3C are shown. D) Representative images of *ss3/4*, *pgm*, *ss3/4/pgm* and WT seedlings after heat treatment started at ZT-1 and ZT8 eight days after heat treatment. E) Expression of cluster 13-1-1 from Figure 1B in a second replicate of mock (ethanol), DCMU and DBMIB treated plants at 22°C and 27°C, treated at ZT-1 and sampled at ZT1. Clusters shown are from the first replicate shown in Figure 4C. F) Average binding of HSFA1a to genes with increased expression after DBMIB and decreased expression after DCMU treatments (intersection from Figure 4B) after treatment at ZT-1 with DCMU (30μM), DBMIB (50μM) and H_2O_2 (5mM). Plants were sampled at ZT1. Replicate of the ChIP-seq experiment shown in Figure 4F. G) Expression of *HSP70*, assayed by qRT-PCR, in plants grown at 22°C short days and either mock treated or treated with 5 mM H_2O_2 at ZT-1 and sampled at ZT1. Error bars are the range of two measurements.

Table S1. Genotyping and qRT-PCR primers used in this study (related to Figures 1, 2, 3 and 4)

**Gene**	**T-DNA mutant**	**Genotyping F primer**	**Genotyping R primer2**	**T-DNA primer**
Unknown Protein (UP1) (AT5G08540)	*up1-1* (Salk 100294)	AGGAAAGCTGGAACAGTGCAT	AGCAGATTCTCTCAAGTACAAGT	SALK_LB1.3 - ATTTTGCCGATTTCGG AAC
STARCH SYNTHASE 4 (SS4) (AT4G18240)	*ss4-1* (Gabi-Kat 290D11)	CGGCTCGAAAAGTCTGATGC	ACCATGCAATGCTTCTATTTCGG	GK_LB - CCCATTTGGACGTGAA TGTAGACAC
	ss4-3 (Salk 096130)	CGGCTCGAAAAGTCTGATGC	ACCATGCAATGCTTCTATTTCGG	SALK_LB1.3
ClpC1 (AT5G50920)	*clpc1-1* (Salk 014058)	CGAAACTGGCTGAGGAGGTAG	TAGTTTCAGGGACATCGCCAC	SALK_LB1.3
**Gene**	**qPCR F primer**	**qPCR R primer**		
UBC22 (AT5G25760)	TCCTCTTAACTGCGACTCAGG	GCGAGGCGTGTATACATTTG		
APA1 (AT1G11910)	CTCCAGAAGAGTATGTTCTGAAAG	TCCCAAGATCCAGAGAGGTC		
APX3 (AT4G35000)	GCCGTGAGCTCCGTTCTCT	TCGTGCCATGCCAATCG		
HSP70 (AT3G12580)	CTGACAGCGAGCGTCTCAT	GGATCACTGTATCTTCTTCCGATT		
