# Supplementary material for: Chloroplast Signaling Gates Thermotolerance in Arabidopsis
Source: Cell Rep. 2018 Feb 13;22(7):1657–65. doi: 10.1016/j.celrep.2018.01.054 (PMC5847188; doi:10.1016/j.celrep.2018.01.054)
Supplement: Document S2. Article plus Supplemental Information [file mmc5.pdf]

Cell Reports

Report

Chloroplast Signaling Gates Thermotolerance in *Arabidopsis*

Graphical Abstract

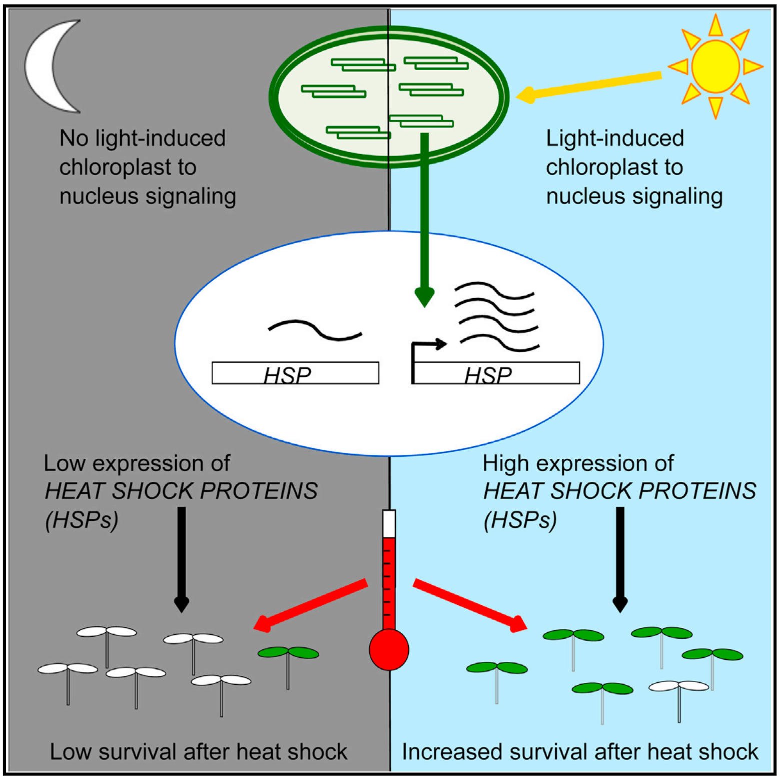

Authors

Patrick J. Dickinson, Manoj Kumar, Claudia Martinho, ..., Ralph Bock, Katja E. Jaeger, Philip A. Wigge

Correspondence

philip.wigge@slcu.cam.ac.uk

In Brief

Plants are most resilient to heat stress during the day, a response controlled by HSFA1 transcription factors activating heat shock genes. Dickinson et al. find that perturbations of chloroplast electron transport affect heat shock gene expression. They show that HSFA1 activity is gated by a light-dependent chloroplast signal.

Highlights

- There is a diurnal pattern of basal thermotolerance in *Arabidopsis*
- Thermotolerance correlates with diurnal expression patterns of heat-associated genes
- Chloroplast mutants have greater heat shock gene expression and thermotolerance
- A chloroplast generated light signal gates HSFA1 and heat shock gene expression

Data and Software Availability

GSE96041

Dickinson et al., 2018, Cell Reports *22*, 1657–1665
February 13, 2018 © 2018 The Author(s).
https://doi.org/10.1016/j.celrep.2018.01.054

CellPress

Cell Reports
Report

OPEN ACCESS
CellPress

Chloroplast Signaling Gates Thermotolerance in *Arabidopsis*

Patrick J. Dickinson,[1,5] Manoj Kumar,[1,2] Claudia Martinho,[1,5] Seong Jeon Yoo,[1] Hui Lan,[1] George Artavanis,[1] Varodom Charoensawan,[1,3] Mark Aurel Schöttler,[4] Ralph Bock,[4] Katja E. Jaeger,[1] and Philip A. Wigge[1,6,*]
[1]Sainsbury Laboratory, University of Cambridge, Cambridge, UK
[2]Department of Plant Molecular Biology, University of New Delhi, Delhi, India
[3]Department of Biochemistry, Faculty of Science, and Integrative Computational BioScience (ICBS) Center, Mahidol University, Bangkok 10400, Thailand
[4]Max-Planck-Institut für Molekulare Pflanzenphysiologie, Potsdam-Golm, Germany
[5]Present address: Department of Plant Sciences, University of Cambridge, Cambridge, UK
[6]Lead Contact
*Correspondence: philip.wigge@slcu.cam.ac.uk
https://doi.org/10.1016/j.celrep.2018.01.054

SUMMARY

Temperature is a key environmental variable influencing plant growth and survival. Protection against high temperature stress in eukaryotes is coordinated by heat shock factors (HSFs), transcription factors that activate the expression of protective chaperones such as *HEAT SHOCK PROTEIN 70* (*HSP70*); however, the pathway by which temperature is sensed and integrated with other environmental signals into adaptive responses is not well understood. Plants are exposed to considerable diurnal variation in temperature, and we have found that there is diurnal variation in thermotolerance in *Arabidopsis thaliana*, with maximal thermotolerance coinciding with higher *HSP70* expression during the day. In a forward genetic screen, we identified a key role for the chloroplast in controlling this response, suggesting that light-induced chloroplast signaling plays a key role. Consistent with this, we are able to globally activate binding of HSFA1a to its targets by altering redox status *in planta* independently of a heat shock.

INTRODUCTION

Temperature has a major role in plant growth and survival and therefore affects crop productivity. For example, wheat yields are predicted to decrease by ~6% for every 1°C rise in global temperature (Asseng et al., 2014). High temperatures induce the expression of protective chaperones and modulate growth responses. Key players in the heat protection response are transcription factors of the HEAT SHOCK FACTOR A1 (HSFA1) family, which trigger the depletion of repressive H2A.Z-nucleosomes and target gene expression (Cortijo et al., 2017; Kumar and Wigge, 2010; Liu et al., 2011), however, the pathways that activate the HSFA1 class TFs and how these perceive temperature and integrate it with other environmental signals are not fully understood. Plants are exposed to considerable diurnal temperature variation and have evolved pathways to anticipate likely future conditions. For example, the cold response pathway is gated by the circadian clock, enabling the degree of responsiveness to be controlled in the context of the environment (Dodd et al., 2006; Lee and Thomashow, 2012) and genes promoting elongation growth and flowering in response to warm, non-stressful, temperature are induced during the night via thermosensory phytochromes (Jung et al., 2016; Legris et al., 2016). A diurnal pattern of thermotolerance has been reported in numerous plant species, including cereals (Laude, 1939), spinach (Li and Guy, 2001), plants using crassulacean acid metabolism (CAM) (Kappen and Lösch, 1984), and black spruce (Colombo et al., 1995). In this study, we find a diurnal pattern of thermotolerance in *Arabidopsis,* which correlates with the expression of *HSP70* over a diurnal cycle. A forward genetic screen with an *HSP70-LUCIFERASE* reporter line revealed a central role for light-induced chloroplast signaling in gating the level of response to high temperature that accounts for diurnal variation in thermotolerance.

RESULTS AND DISCUSSION

Thermotolerance Varies Diurnally and Correlates with the Expression of *HSPs*

To investigate whether the time of day gates thermotolerance in *Arabidopsis*, plants were grown for 7 days at constant 17°C, 22°C, or 27°C and survival after a heat treatment applied at nine points over a 24-hr time course was measured. We found that *Arabidopsis* has a diurnal pattern of thermotolerance (Figures 1A and S1A), consistent with reports from other plant species (Colombo et al., 1995; Kappen and Lösch, 1984; Laude, 1939; Li and Guy, 2001). The lowest levels of survival occur shortly before dawn and greatest survival occurs during the light period. The growth temperature affects the levels and patterns of thermotolerance, with higher growth temperatures resulting in increased thermotolerance occurring earlier in the day compared to lower temperatures (Figure 1A).

To identify gene expression changes underlying the diurnal pattern of thermotolerance, we analyzed transcriptomes of plants grown at constant 17°C, 22°C, and 27°C over a 24-hr time course. Genes were clustered based on expression patterns and a prominent cluster (cluster 13-1-1) showing a temperature responsive morning peak of expression was identified

This is an open access article under the CC BY license (http://creativecommons.org/licenses/by/4.0/).

OPEN ACCESS
CellPress

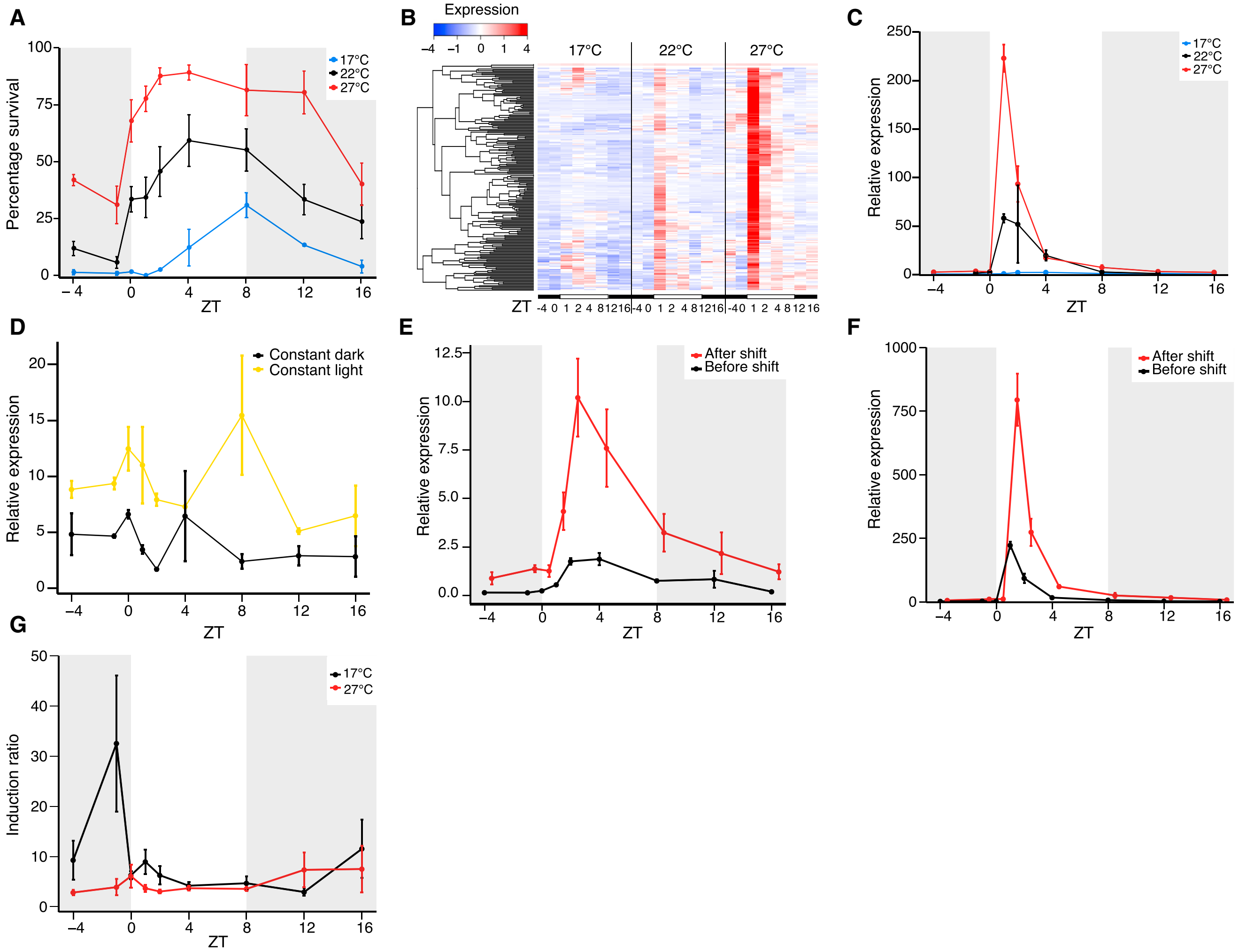

Figure 1. A Diurnal Pattern of Basal Thermotolerance Correlates with a Diurnal Pattern of Heat-Associated Gene Expression

(A) Basal thermotolerance of WT seedlings over a diurnal time course. Error bars are ±SEM (n = 3), where 81 seedlings were scored per time point and temperature for three independent experiments.

(B) Heatmap of the expression of genes in cluster 13-1-1. Transcriptomes shown from plants grown in short days (8 hr light, 16 hr dark) at constant 17°C, 22°C, or 27°C. Black bars below heatmap indicate samples taken in the dark (ZT−4, ZT0, ZT12, and ZT16) and white bars indicate samples taken in the light (ZT1, ZT2, ZT4, and ZT8). In heat maps, low to high expression (*Z* scores) is shown as blue to red.

(C–F) Expression of *HSP70* in WT seedlings assayed by qRT-PCR.

(C) Plants grown at 17°C, 22°C, and 27°C. Error bars are ± the range of two measurements (n = 2) or SEM (n = 3 to 4).

(D) Seedlings entrained in short days at 27°C and shifted to constant light or constant dark at ZT8 of the seventh day after germination. Plants were sampled starting at ZT−4 on the eighth day. Error bars are ± the range of two measurements.

(E and F) Before and after a 30-min shift to 45°C in seedlings grown at 17°C (E) and 27°C (F).

(G) Induction ratios (expression after shift/expression before shift) of *HSP70* expression after the temperature shifts in (F) and (G). Error bars are ±SEM (n = 3).

(Figure 1B). This morning cluster is strongly enriched for genes associated with the response to heat, high light, and oxidative stress (Table S2), including a number of *HEAT SHOCK PROTEINS* (*HSPs*) required for thermotolerance, such as *HSP101* (Queitsch et al., 2000), consistent with this cluster contributing to the diurnal pattern of thermotolerance. The promoters of genes in the morning cluster show enrichment for HSF binding sites known as heat shock elements (HSE) (Schöffl et al., 1998) (Table S3), suggesting that the temperature responsiveness of this cluster is HSF-dependent. Because *HSP70* expression is an indicator of temperature perception status (Kumar and Wigge, 2010) and is representative of the morning cluster (Figures 1C and S1B), we further analyzed *HSP70* to understand how the expression of this cluster of genes is controlled.

To test whether the diurnal pattern of *HSP70* expression is controlled by the circadian clock, plants were entrained in short days at constant 27°C before shifting to constant light or constant dark. Compared to plants grown at constant 27°C in short days (Figure 1C) there was an absence of the morning peak of *HSP70* expression in both constant light and constant dark (Figure 1D) indicating that the diurnal expression pattern is not primarily controlled by the circadian clock, consistent with work

performed in spinach (Li and Guy, 2001). The morning peak was present, albeit substantially lower, in a *CIRCADIAN CLOCK ASSOCIATED 1* (*CCA1*) overexpressing line (*CCA1:OX*) (Figure S1C), which has an arrhythmic clock (Wang and Tobin, 1998). The lower morning peak in *CCA1:OX* may be the result of an indirect effect of altered reactive oxygen species (ROS) signaling in *CCA1:OX* (Lai et al., 2012). Supporting the idea that light regulation is dominant over circadian regulation of *HSP70* expression, the morning peak of LUCIFERASE activity in a line containing an *HSP70-LUCIFERASE* (*HSP70-LUC*) reporter (Kumar and Wigge, 2010) occurs 1 hr after lights come on when the night is extended by up to 4 hr (Figure S1D). The morning peak of *HSP70* expression is lost in both constant light and constant dark (Figure 1D) suggesting that it is dependent on the perception of the dark to light transition. The diurnal pattern of *HSP70* expression was present in *cryptochrome* and *phytochrome* null mutants and plants with a constitutively active phytochrome *(cry1/2*, *phyABCDE*, and *phyYHB*) (Jung et al., 2016) (Figures S1C and S1E) indicating that the morning peak is independent of the main blue (cryptochrome) and red/far red (phytochrome) photoreceptors, although the involvement of other photoreceptors has not been ruled out.

Because there is a diurnal pattern of *HSP70* expression at constant temperature (Figures 1C and S1B), we investigated whether the time of day also affects the induction of *HSP70* by heat. *HSP70* expression was assayed in plants grown at 17°C or 27°C and shifted to 45°C for 30 min, replicating the conditions of the thermotolerance assays. Levels of *HSP70* expression after a shift had a similar pattern to *HSP70* expression at constant temperature (Figures 1E and 1F) and this correlated with the diurnal patterns of thermotolerance (Figure 1A). The induction of *HSP70* by heat was quantified as the expression after shift divided by the expression before shift. Although there was large variability in *HSP70* expression levels between time points and growth conditions, induction ratios were similar (Figure 1G). The induction of *HSP70* expression in plants grown at 17°C and shifted at Zeitgeber time (ZT)−1 was higher, but this likely reflects experimental noise as the expression levels before the shift at ZT−1 are so low they are hard to measure accurately. There were also no large differences between induction ratios of plants grown at constant 17°C and 27°C, despite large differences in expression levels (Figures 1E–1G). The data are consistent with a model where light gates the magnitude of the response to high temperature, with expression after a temperature shift being a multiple of the expression before a shift. The expression of *HSP70* is very low in the dark, and consequently its expression at higher temperature is also minimal. By comparison, in the light a higher baseline expression of *HSP70* coincides with a proportionately higher expression level following induction. In this way, light can be thought as "priming" the heat shock response.

Nuclear Genes Encoding Chloroplast Proteins Are Necessary for Correct HSP70 Expression during the Day, and This Correlates with Thermotolerance

To investigate how the diurnal pattern of thermotolerance might be controlled, we screened for mutants with altered *HSP70* expression in the morning using the *HSP70-LUC* reporter line. Two mutant lines, 429 and 2641, were identified as having increased LUC activity in the morning at 17°C compared to wild-type (WT) (Figure 2A). The causal mutation in line 429 was mapped to a stop codon in a gene encoding an unknown protein found to be localized in the thylakoid membrane (*AT5G08540*) (Reiland et al., 2011; Simm et al., 2013) and the causal mutation in line 2641 was mapped to a stop codon in *STARCH SYNTHASE 4* (*SS4*) (Figure 2B; Supplemental Experimental Procedures). A T-DNA insertion in the unknown protein (*up1-1*) shows increased *HSP70* expression compared to WT, similar to line 429, and two independent *ss4* T-DNA mutants (*ss4-1* [Roldán et al., 2007] and *ss4-3* [Crumpton-Taylor et al., 2013]) show increased *HSP70* expression compared to WT, similar to line 2641 (Figure 2C). Complementation crosses confirmed that the *HSP70-LUC* mis-expression phenotypes in lines 429 and 2641 were caused by loss of UP1 and SS4 function respectively as F1 plants of crosses between the EMS and T-DNA mutants fail to rescue the LUC activity phenotypes of the EMS lines, whereas crosses to WT do rescue (Figures S2A and S2B).

The *up1* and *ss4* mutants have increased *HSP70* expression at 17°C ZT2 (Figure 2C). To investigate whether increased *HSP70* expression in these mutants is specific to a particular time of day, *HSP70* expression was assayed from ZT−1 to ZT12. This time course showed that the *up1* and *ss4* mutants have greatly increased *HSP70* expression specifically during the day (Figure 2D). In WT plants, the diurnal pattern of *HSP70* expression correlates with basal thermotolerance (Figures 1A and 1C). Similarly, increased *HSP70* expression during the day in *up1* and *ss4* (Figure 2D) correlates with increased thermotolerance in these mutants (Figures 2E and S2C).

The diurnal pattern of *HSP70* expression is light-dependent but broadly circadian clock and photoreceptor independent (Figures 1D and S1C–S1E) necessitating the involvement of another pathway to generate the morning peak of *HSP70* expression and therefore increase thermotolerance in response to light. As well as being the site of photosynthesis, the chloroplast is an environmental sensor and can transmit signals to effect nuclear gene expression through retrograde signaling (Chan et al., 2016), including expression of an *HSP70* gene in *Chlamydomonas* (Kropat et al., 1997). Both *UP1* and *SS4* encode chloroplast-localized proteins suggesting that the chloroplast is involved in generating the morning peak of *HSP70* expression. This is supported by the observation that the morning peak of *HSP70* expression is absent in light-exposed roots (Figure S2D) and that mutations in other genes encoding chloroplast-localized proteins, including *maltose exporter1* (*mex1*) (Niittylä et al., 2004) and *clpc1* (Sjögren et al., 2014) were identified in the screen for *HSP70-LUC* mis-expression and these mutants also have increased *HSP70* expression specifically during the day (Figure S2E).

Increased Expression of Heat-Associated Genes in *ss4* Is Associated with the Light Reactions of Photosynthesis

As the function of *SS4* is well-characterized (Roldán et al., 2007; Crumpton-Taylor et al., 2013; Ragel et al., 2013), we used it to further characterize how chloroplast signaling affects diurnal patterns of heat-associated gene expression and thermotolerance. SS4 is a soluble starch synthase that catalyzes the addition of

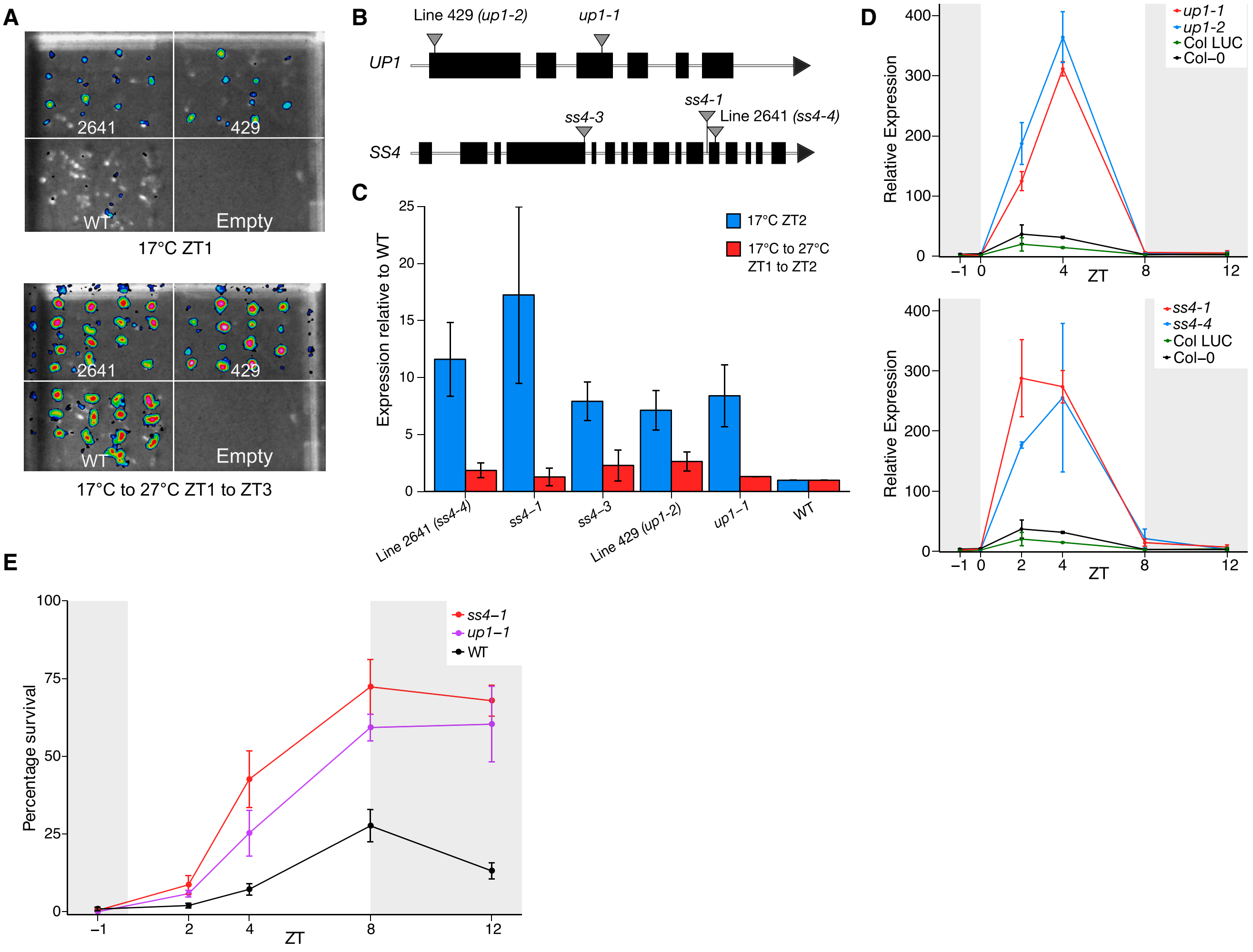

Figure 2. Mutations in Genes Encoding Chloroplast-Localized Proteins Cause Increased *HSP70* Expression and Increased Thermotolerance

(A) False color image of LUCIFERASE activity of seedlings imaged at 17°C ZT1 and after a shift from 17°C to 27°C from ZT1 to ZT3. Two mutant lines, 2641 and 429, and WT (col-0 containing *pHSP70::LUC*), are shown from the same plate.

(B) Schematics of *UP1* and *SS4* with the position of mutations shown. Line 429 (*up1-2*) has a C to T substitution on chromosome (chr) V at locus 2763941 mutating Gln10 to a stop codon. Line 2641 (*ss4-4*) has a C to T substitution on chr IV at locus 10086122 mutating Gln872 to a stop codon.

(C and D) *HSP70* expression assayed by qRT-PCR.

(C) Mutants at 17°C ZT2 and after a shift from 17°C to 27°C from ZT1 to ZT2. Expression shown relative to WT sampled from the same plate. Error bars are ± the range of two measurements (n = 2) or SEM (n = 3 to 4).

(D) *up1* and *ss4* mutants over a time course at 17°C in short days sampled at ZT−1, ZT0, ZT2, ZT4, ZT8, and ZT12. Error bars are ± the range of two measurements (n = 2) or SEM (n = 3).

(E) Thermotolerance of *up1-1*, *ss4-1*, and WT with plants grown at 17°C and treated at ZT−1, ZT2, ZT4, ZT8, and ZT12. Error bars are ±SEM (n = 3).

glucose from ADP-glucose to amylopectin chains (Streb and Zeeman, 2012) (Figure 3A). There are four soluble starch synthases in *Arabidopsis* and *ss4* is the only single mutant with a clear phenotype, likely because of its involvement in initiating starch granule synthesis (Crumpton-Taylor et al., 2013).

Expression of *HSP70* in *ss4* at 17°C shows the strongest difference to WT in the morning (Figure 2D), suggesting that the *ss4* mutant may affect the regulation of the morning cluster of genes (Figure 1B). To confirm this, we analyzed the *ss4* transcriptome over a diurnal time course at 17°C. Differentially expressed genes between *ss4* and WT were defined using DESeq2 (Love et al., 2014) and clustered based on expression patterns (Figures 3B and S3A). A cluster of genes (cluster 4) showing increased expression in *ss4* in the morning was strongly enriched for heat-associated genes (Table S2). This cluster overlapped with the cluster of morning peak genes (Figure S3B), and HSEs were enriched in the promoters of genes in this cluster (Table S3).

Defective starch synthesis leads to over-accumulation of ADP-glucose in *ss4* and *ss3/4* mutants (Ragel et al., 2013). This disrupts the photosynthetic electron transport (PET) chain as the over-accumulation of ADP-glucose limits the availability of ADP for the chloroplast ATP-synthase. This substrate-limitation results in decreased proton eflux from the thylakoid lumen through ATP synthase and thus an over-acidified lumen (Sharkey

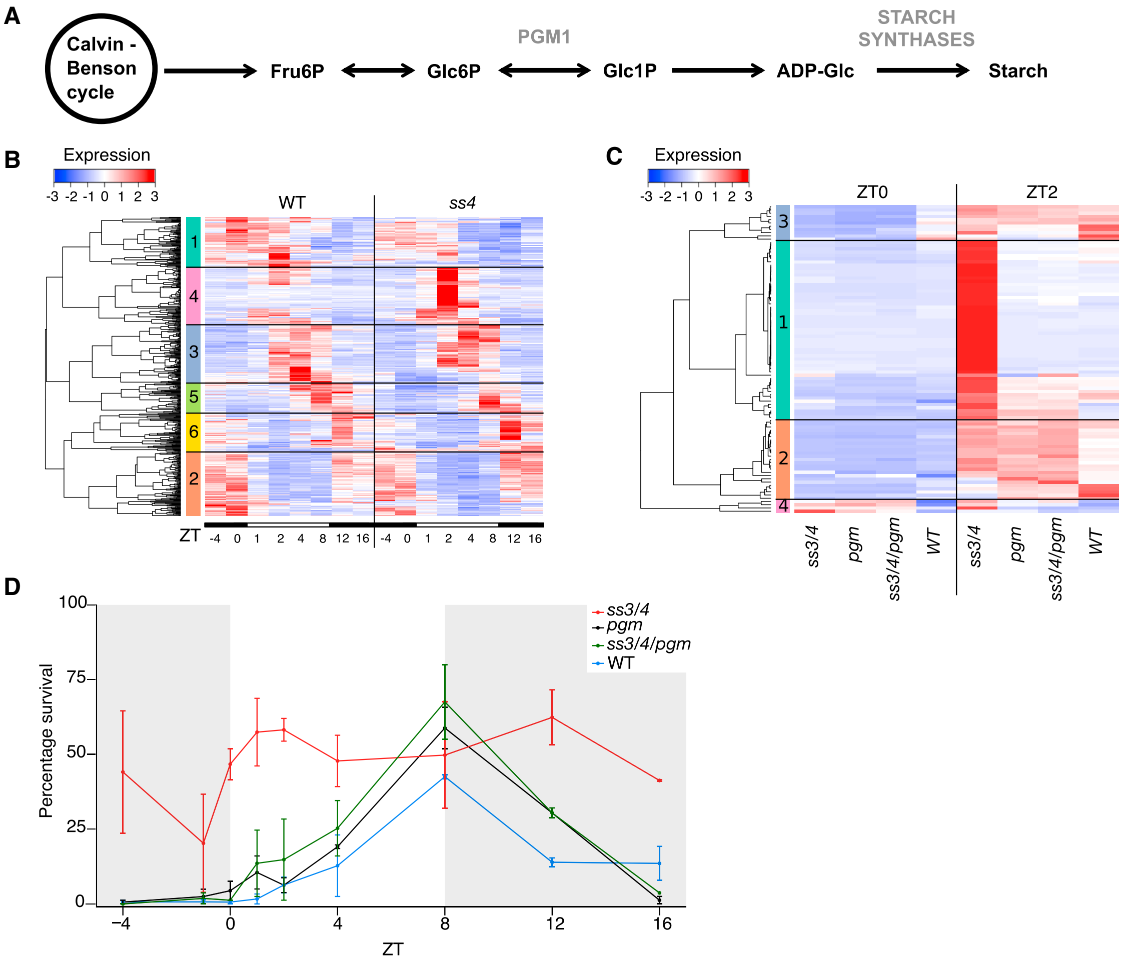

Figure 3. Increased Expression of Heat-Associated Genes in *ss4* Is Caused by Alterations to the Light Reactions of Photosynthesis

(A) Simplified schematic of the starch synthesis pathway.

(B) Heatmap of the expression of genes differentially expressed between *ss4-1* and WT at any sampled time-point. Transcriptomes are shown from plants grown in short days at 17°C. Black bars underneath heatmap show samples taken in the dark (ZT−4, ZT0, ZT12, and ZT16) and white bars show samples taken in the light (ZT1, ZT2, ZT4, and ZT8). Replicate shown in Figure S3A.

(C) Heatmap of expression of cluster 4 from (B) in *ss3/4*, *pgm*, *ss3/4/pgm*, and WT (col-0) at 17°C ZT0 and ZT2. Replicate shown in Figure S3C. (B and C) Clusters shown on the left of heat maps and low to high expression (*Z* scores) is shown as blue to red.

(D) Basal thermotolerance of *ss3/4*, *pgm*, *ss3/4/pgm*, and WT (col-0) grown at 17°C in short days and treated at ZT−4, ZT−1, ZT0, ZT1, ZT2, ZT4, ZT8, ZT12, and ZT16. Error bars are the range of two measurements.

and Vanderveer, 1989; Takizawa et al., 2008). As a consequence, plastoquinol re-oxidation at the cytochrome b6f complex is slowed down (“photosynthetic control”) and non-photochemical quenching of excitation energy in the PSII antenna bed is strongly induced, something that has been suggested to initiate retrograde signaling cascades (Rott et al., 2011).

The *ss4* mutation affects both the carbon and light reactions of photosynthesis (Ragel et al., 2013) and causes increased expression of heat-associated genes in response to light (Figure 3B; Table S2). To determine whether the overexpression of heat-associated genes in *ss4* is associated with the carbon or light reactions of photosynthesis, we assessed the expression of cluster 4 (Figure 3B) in the transcriptomes of *ss3/4*, *plastidal phosphoglucomutase* (*pgm*), *ss3/4/pgm*, and WT at ZT0 and ZT2 at 17°C. Four clusters were defined and the genes in the largest cluster (cluster 4-1) were strongly overexpressed in *ss3/4* compared to WT in the morning (ZT2). This overexpression was absent in *pgm* and *ss3/4/pgm* (Figures 3C and S3C). Cluster 4-1 is strongly enriched for heat-associated genes and for genes with HSEs in their promoters (Table S3). *pgm* has very low levels of starch and increased levels of soluble sugars compared to WT (Ragel et al., 2013; Streb et al., 2009), therefore, effects on the carbon reactions do not cause increased expression of heat-associated genes as these genes are expressed similarly to WT in *pgm* (Figures 3C and S3C). Phenotypes associated with the light reactions such as photochemical efficiency, quantum yield of photosystem two, and an over-reduction of the plastoquinone (PQ) pool, are strongly affected in *ss4* and *ss3/4* compared to *pgm* (Ragel et al., 2013) suggesting that the mis-expression of cluster 4-1 in *ss4* and *ss3/4* is caused by alterations

to the light reactions. Interestingly, the overexpression of cluster 4-1 is rescued in an *ss3/4/pgm* mutant, in which the over-accumulation of ADP-glucose in *ss3/4* is suppressed (Ragel et al., 2013), suggesting that the over-accumulation of ADP-glucose and the subsequent effects on the light reactions are the cause of the overexpression of heat-associated genes in *ss4* and *ss3/4.* The expression patterns of heat-associated genes in *ss3/4*, *pgm*, and *ss3/4/pgm* correlate with thermotolerance (Figures 3D and S3D) consistent with the central role of the light-induced morning cluster in determining thermotolerance.

The Morning Peak of Heat-Associated Gene Expression Is Associated with a Reduction of the PQ Pool

Diurnal patterns of heat-associated gene expression and thermotolerance are affected by the light reactions of photosynthesis and the redox state of the PQ pool has been shown to affect the expression of heat-associated genes in response to excess light (Jung et al., 2013). Because of this and the observation that the PQ pool is more reduced in *ss4* than WT, and that this over-reduction is rescued in *ss3/4/pgm* (calculated using data from Ragel et al. [2013]; Supplemental Experimental Procedures), we investigated whether the redox state of the PQ pool affects *HSP70* expression. The redox state of the PQ pool can be modified experimentally by the application of 3-(3,4-dichlorophenyl)-1,1-dimethylurea (DCMU) and 2,5-dibromo-3-methyl-6-isopropyl-p-benzoquinone (DBMIB). DCMU prevents plastoquinol binding to Q_B and thus blocks electron transfer to the PQ pool, resulting in a more oxidized PQ pool (Pfalz et al., 2012). DBMIB prevents the PQ pool from transferring electrons to the cytochrome b_6f (cyt-b_6f) complex, resulting in a reduced PQ pool (Pfalz et al., 2012). We see that the morning peak of *HSP70* expression is abolished by DCMU, while it is increased by DBMIB, indicating that a reduced PQ pool is required for *HSP70* induction in the morning (Figure 4A).

As DCMU and DBMIB treatments oppositely affect *HSP70* expression, we examined which genes were affected in a similar way, as the expression of these genes may also be affected by the redox state of the PQ pool. At 22°C DBMIB treatment resulted in the overexpression of 4,298 genes and DCMU treatment resulted in the under-expression of 1,508 genes (Figure 4B). Despite the chemical treatments resulting in large changes to the transcriptome, only a small set of genes were both overexpressed after DBMIB and under-expressed after DCMU treatment (Figure 4B). This set of genes was strongly enriched for genes associated with responses to heat (Table S2) and for genes with HSEs in their promoters (Table S3) suggesting that the redox state of the PQ pool affects the morning peak of heat-associated gene expression. This is supported by the observation that the expression of the majority of genes in the morning cluster (Cluster 13-1-1 from Figure 1B) are increased by DBMIB treatment and decreased by DCMU treatment (Figure 4C and S3E). Similarly, the majority of genes that are oppositely affected by DCMU and DBMIB treatment have a morning peak of expression in untreated samples (Figure 4D).

As a reduced PQ pool is associated with the morning peak of heat-associated gene expression, we predicted that the PQ pool would be reduced by light in the morning. As expected the PQ pool was fully oxidized during the night, based on an indirect measure of the redox state of the PQ pool, qL (Kramer et al., 2004). With the onset of light qL decreased and then rose throughout the first hour of the day (Figure 4E), reflecting a reduction of the PQ pool by light and a subsequent re-oxidation when the Calvin-Benson cycle became light-activated. qL values decreased to a lower level at 17°C compared to 27°C and the amount of time until qL returned to one was slower at 17°C compared to 27°C, likely due to a slower induction of the Calvin-Benson cycle (Figure 4E). This indicates that the PQ pool becomes more reduced by light at lower temperatures; therefore, the level of reduction of the PQ pool does not appear to transmit temperature information in the ambient range. In *Chlamydomonas*, it has been proposed that light and temperature signals are integrated independently via Mg-protoporphyrin IX (MgProto) and HSFs, respectively (von Gromoff et al., 2006), however, the role of MgProto in retrograde signaling has been questioned (Moulin et al., 2008; Mochizuki et al., 2008). Because HSFs have previously been shown to respond to redox status (Jung et al., 2013; Miller and Mittler, 2006; Volkov et al., 2006), we investigated whether they might integrate both light and heat signals for morning cluster gene expression in *Arabidopsis*. The HSFA1 clade of HSFs have been shown to be essential for the early transcriptional response to heat (Liu et al., 2011), so we investigated the binding of HSFA1a to genes overexpressed after DBMIB treatment and decreased after DCMU treatment (intersection of Figure 4B) by chromatin immunoprecipitation sequencing (ChIP-seq). Interestingly, we observe a strong induction of HSFA1a binding to these genes in response to DBMIB at 22°C, while DCMU prevents the binding of HSFA1a to these genes (Figures 4F and S3F). Taken together, these results are consistent with a model where HSFA1a is able to integrate both the light and temperature signals, with the light signal being either PQ redox state or associated H_2O_2. Direct H_2O_2 transfer from the chloroplast to nucleus has been shown to be involved in high light signaling and was repressed by DCMU treatment (Exposito-Rodriguez et al., 2017). As H_2O_2 can activate HSFs (Volkov et al., 2006) and cause increased *HSP70* expression (Figure S3G), a model where H_2O_2 is produced in the chloroplast at the onset of light and transferred to the nucleus to activate the expression of heat-associated genes is an attractive model for generating diurnal patterns of heat-associated gene expression and thermotolerance.

Conclusions

We find a diurnal pattern of thermotolerance in *Arabidopsis* that is a consequence of a gating effect of light-induced chloroplast signaling on the expression levels of heat-associated genes such as *HSP70*, accounting for a long-standing observation that plants have a differential ability to withstand heat stress in the day and night (Laude, 1939).

Under constant temperature conditions, the expression of morning peak genes such as *HSP70* is repressed into the afternoon (Figures 1B and 1C), likely because of repressive interactions of HSP70 and HSP90 with HSFA1s (Ohama et al., 2016) resulting in a negative feedback loop. The sharpness of the morning peak may also be influenced by growth chambers, with plants being subjected to a sudden change from darkness to full light, while plants in the field will experience a more gradual rise in light levels. Protective chaperones such as HSP70 have ATPase activity, and

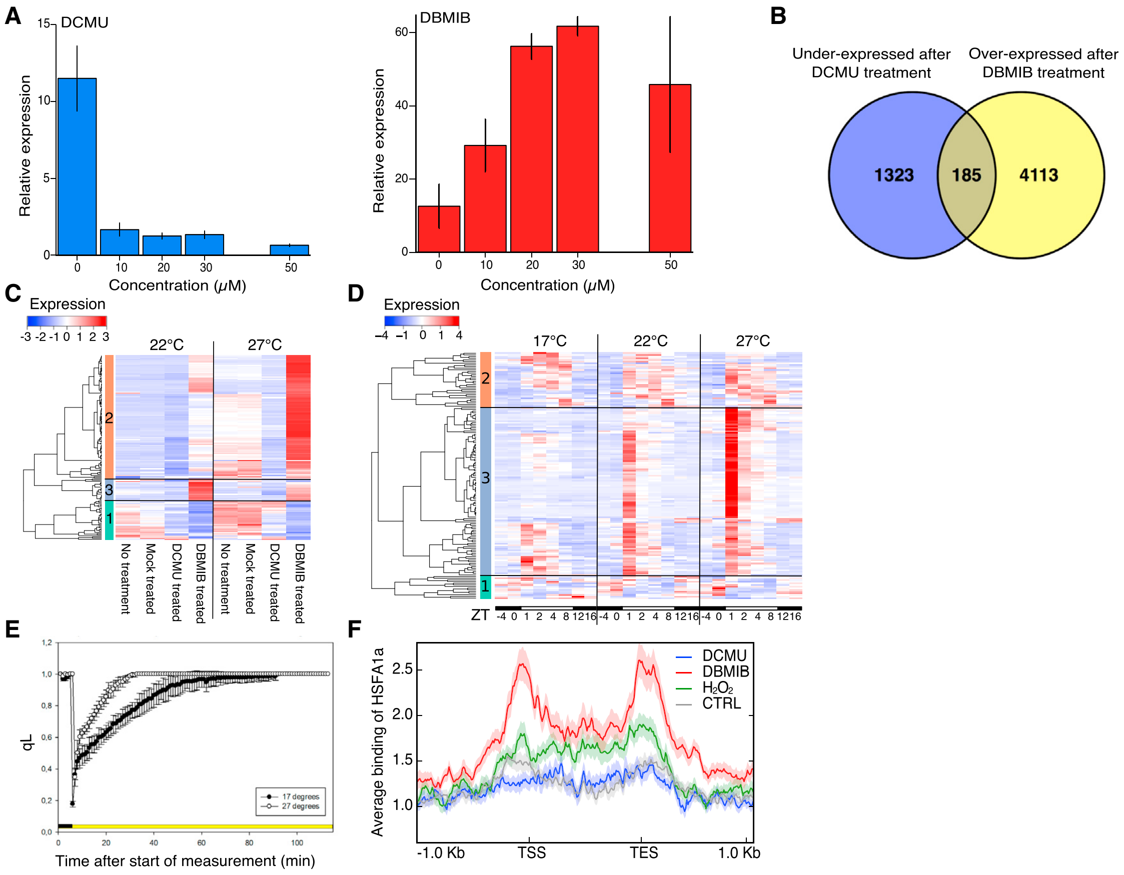

Figure 4. The Redox State of the Plastoquinone Pool Is Associated with the Expression of the Morning Cluster of Heat-Associated Genes

(A) *HSP70* expression assayed by qRT-PCR after DCMU and DBMIB treatment of seedlings grown at 22°C in short days. Plants treated at ZT−1 and sampled at ZT1. Error bars are ±SEM (n = 3).

(B) Overlaps of genes under-expressed (Log2 fold change <−0.5) after DCMU (30 μM) and overexpressed (Log2 fold change >0.5) after DBMIB (50 μM) treatments compared to a mock treatment at 22°C, treated at ZT−1 and sampled at ZT1.

(C) Expression of cluster 13-1-1 (from Figure 1C) in transcriptomes of untreated, mock-treated, DCMU, and DBMIB-treated seedlings at 22°C and 27°C ZT1 after treatment at ZT−1. Replicate shown in Figure S3E.

(D) Expression of the genes under-expressed after DCMU treatment and overexpressed after DBMIB treatment (overlap from B) in WT over a 24-hr time course (same as used for Figure 1C) at 17°C, 22°C, and 27°C. Black bars below heatmap indicate samples taken in the dark (ZT−4, ZT0, ZT12, and ZT16) and white bars indicate samples taken in the light (ZT1, ZT2, ZT4, and ZT8). (C and D) Clusters are shown on the left of heatmap and low to high expression (*Z* scores) is shown as blue to red.

(E) qL over dawn at 17°C and 27°C. Error bars are ±SD (n = 4).

(F) Average binding of HSFA1a to genes from the intersection of Figure 4B after treatment at ZT−1 with DCMU (30 μM), DBMIB (50 μM), H_2O_2 (5 mM), and a mock ethanol treatment. Plants sampled at ZT1. Shading represents SE. Replicate shown in Figure S3F.

at high expression levels, can represent a significant energy demand. *Drosophila* cells overexpressing *HSP70* show greatly attenuated growth (Feder et al., 1992), and constitutive activation of heat-associated gene expression in *Arabidopsis* likewise greatly reduces growth (Ogawa et al., 2007). It may therefore be advantageous to limit heat-associated gene expression to the daytime, when temperature stress is most probable.

EXPERIMENTAL PROCEDURES

Plant Material and Growth Conditions

Growth conditions were often specific to the experiment performed and these are described in the Supplemental Experimental Procedures.

For details of mutant lines used in this study see the Supplemental Experimental Procedures and Table S1.

Thermotolerance Assays

A thermotolerance assay was developed based on a previously described protocol (Silva-Correia et al., 2014). After 7 days of growth, plates were floated in a water bath preheated to 45°C for 30 min. Survival was defined as the ability to produce new green leaves.

Gene Expression Analysis

For qRT-PCR assays, RNA was extracted using a previously described phenol:chloroform extraction method (Box et al., 2011). Transcript levels were quantified using SybrGreen (Roche) and samples assayed with technical triplicates and on a LightCycler 480 (Roche).

Identifying Candidate Mutants and Mapping Causal Genes

A previously described fusion between the promoter of *HSP70* and *LUCIFERASE* (*pHSP70::LUC*) (Kumar and Wigge, 2010) was used to screen EMS mutagenized Col-0 plants. LUC activity was imaged using a Photek

HRPCS218 camera. Plates were imaged at 17°C at ZT1 and shifted to 27°C for 2 hr and imaged again at ZT3. Causal genes in were mapped by sequencing F2 plants displaying mutant phenotypes after outcrossing to *Landsberg erecta.*

Transcriptomics

The MagMax RNA extraction kit (Ambion) was used to extract RNA from 20–25, 8-day-old seedlings and libraries prepared using the Illumina TruSeq stranded kit. Sequencing was performed on a NextSeq500 (Illumina) and an in-house RNA sequencing (RNA-seq) processing pipeline was used to process sequence data.

ChIP-Seq

Chromatin was extracted from 1 g of cross-linked plant material, fragmented by sonication and ChIP was performed using anti-FLAG M2 magnetic beads (Sigma, M8823) coupled to a 1/1 mix of protein-A and protein-G Dynabeads (Life Technologies, 10001D and 10003D). ChIP-seq libraries were prepared using a TruSeq ChIP Library kit (Illumina) and sequenced on a NextSeq 500 (Illumina).

Chlorophyll Fluorescence

The Maxi version of the Imaging-PAM M-series (Walz) was used to determine chlorophyll-a fluorescence parameters of intact plants. A custom-made version of the 3010-GWK1 gas exchange chamber (Walz) was used to control humidity and temperature.

DATA AND SOFTWARE AVAILABILITY

The accession number for the raw and processed data from the RNA sequencing experiments reported in this paper is GEO: GSE96041.

SUPPLEMENTAL INFORMATION

Supplemental Information includes Supplemental Experimental Procedures, three figures, and four tables and can be found with this article online at https://doi.org/10.1016/j.celrep.2018.01.054.

ACKNOWLEDGMENTS

We thank Ángel Mérida, Sam Zeeman, and Benoit Landrien for Arabidopsis strains. We thank members of the Wigge laboratory for feedback and discussions. P.J.D. was supported by a Gatsby PhD Studentship (PTDC.GAAB). This work was supported by a grant from the Biotechnology and Biological Sciences Research Council (BB/I013350/1). The P.A.W. laboratory is supported by a Fellowship from the Gatsby Foundation (GAT3273/GLB).

AUTHOR CONTRIBUTIONS

P.J.D. conceived and performed experiments and wrote the manuscript. M.K., C.M., S.J.Y., G.A., and K.E.J. performed experiments. H.L. and V.C. used bioinformatics to analyze results, and M.A.S. and R.B. contributed experiments and analysis. P.A.W. conceived of the project and contributed to writing the manuscript and analyzing the data.

DECLARATION OF INTERESTS

The authors declare no competing interests.

Received: March 17, 2017
Revised: November 17, 2017
Accepted: January 18, 2018
Published: February 13, 2018

REFERENCES

Asseng, S., Ewert, F., Martre, P., Rötter, R., Lobell, D., Cammarano, D., Kimball, B.A., Ottman, M., Wall, G., White, J., et al. (2014). Rising temperatures reduce global wheat production. Nat. Clim. Chang. *5*, 143–147.

Box, M.S., Coustham, V., Dean, C., and Mylne, J.S. (2011). Protocol: a simple phenol-based method for 96-well extraction of high quality RNA from Arabidopsis. Plant Methods *7*, 7.

Chan, K.X., Phua, S.Y., Crisp, P., McQuinn, R., and Pogson, B.J. (2016). Learning the languages of the chloroplast: retrograde signaling and beyond. Annu. Rev. Plant Biol. *67*, 25–53.

Colombo, S.J., Timmer, V.R., Colclough, M.L., and Blumwald, E. (1995). Diurnal variation in heat tolerance and heat shock protein expression in black spruce (*Piceamariana*). Can. J. For. Res. *25*, 369–375.

Cortijo, S., Charoensawan, V., Brestovitsky, A., Buning, R., Ravarani, C., Rhodes, D., van Noort, J., Jaeger, K.E., and Wigge, P.A. (2017). Transcriptional regulation of the ambient temperature response by H2A.Z nucleosomes and HSF1 transcription factors in Arabidopsis. Mol. Plant *10*, 1258–1273.

Crumpton-Taylor, M., Pike, M., Lu, K.J., Hylton, C.M., Feil, R., Eicke, S., Lunn, J.E., Zeeman, S.C., and Smith, A.M. (2013). Starch synthase 4 is essential for coordination of starch granule formation with chloroplast division during Arabidopsis leaf expansion. New Phytol. *200*, 1064–1075.

Dodd, A.N., Jakobsen, M.K., Baker, A.J., Telzerow, A., Hou, S.W., Laplaze, L., Barrot, L., Poethig, R.S., Haseloff, J., and Webb, A.A.R. (2006). Time of day modulates low-temperature Ca signals in Arabidopsis. Plant J. *48*, 962–973.

Exposito-Rodriguez, M., Laissue, P.P., Yvon-Durocher, G., Smirnoff, N., and Mullineaux, P.M. (2017). Photosynthesis-dependent H2O2 transfer from chloroplasts to nuclei provides a high-light signalling mechanism. Nat. Commun. *8*, 49.

Feder, J.H., Rossi, J.M., Solomon, J., Solomon, N., and Lindquist, S. (1992). The consequences of expressing hsp70 in Drosophila cells at normal temperatures. Genes Dev. *6*, 1402–1413.

Jung, H.S., Crisp, P.A., Estavillo, G.M., Cole, B., Hong, F., Mockler, T.C., Pogson, B.J., and Chory, J. (2013). Subset of heat-shock transcription factors required for the early response of Arabidopsis to excess light. Proc. Natl. Acad. Sci. USA *110*, 14474–14479.

Jung, J.H., Domijan, M., Klose, C., Biswas, S., Ezer, D., Gao, M., Khattak, A.K., Box, M.S., Charoensawan, V., Cortijo, S., et al. (2016). Phytochromes function as thermosensors in Arabidopsis. Science *354*, 886–889.

Kappen, L., and Lösch, R. (1984). Diurnal patterns of heat tolerance in relation to CAM. Z. Pflanzenphysiol. *114*, 87–96.

Kramer, D.M., Johnson, G., Kiirats, O., and Edwards, G.E. (2004). New fluorescence parameters for the determination of QA redox state and excitation energy fluxes. Photosynth. Res. *79*, 209–218.

Kropat, J., Oster, U., Rüdiger, W., and Beck, C.F. (1997). Chlorophyll precursors are signals of chloroplast origin involved in light induction of nuclear heat-shock genes. Proc. Natl. Acad. Sci. USA *94*, 14168–14172.

Kumar, S.V., and Wigge, P.A. (2010). H2A.Z-containing nucleosomes mediate the thermosensory response in Arabidopsis. Cell *140*, 136–147.

Lai, A.G., Doherty, C.J., Mueller-Roeber, B., Kay, S.A., Schippers, J.H.M., and Dijkwel, P.P. (2012). CIRCADIAN CLOCK-ASSOCIATED 1 regulates ROS homeostasis and oxidative stress responses. Proc. Natl. Acad. Sci. USA *109*, 17129–17134.

Laude, H.H. (1939). Diurnal cycle of heat resistance in plants. Science *89*, 556–557.

Lee, C.M., and Thomashow, M.F. (2012). Photoperiodic regulation of the C-repeat binding factor (CBF) cold acclimation pathway and freezing tolerance in *Arabidopsis thaliana*. Proc. Natl. Acad. Sci. USA *109*, 15054–15059.

Legris, M., Klose, C., Burgie, E.S., Rojas, C.C.R., Neme, M., Hiltbrunner, A., Wigge, P.A., Schäfer, E., Vierstra, R.D., and Casal, J.J. (2016). Phytochrome B integrates light and temperature signals in Arabidopsis. Science *354*, 897–900.

Li, Q.B., and Guy, C.L. (2001). Evidence for non-circadian light/dark-regulated expression of Hsp70s in spinach leaves. Plant Physiol. *125*, 1633–1642.

Liu, H.C., Liao, H.T., and Charng, Y.Y. (2011). The role of class A1 heat shock factors (HSFA1s) in response to heat and other stresses in Arabidopsis. Plant Cell Environ. *34*, 738–751.

Love, M.I., Huber, W., and Anders, S. (2014). Moderated estimation of fold change and dispersion for RNA-seq data with DESeq2. Genome Biol. *15*, 550.

Miller, G., and Mittler, R. (2006). Could heat shock transcription factors function as hydrogen peroxide sensors in plants? Ann. Bot. *98*, 279–288.

Mochizuki, N., Tanaka, R., Tanaka, A., Masuda, T., and Nagatani, A. (2008). The steady-state level of Mg-protoporphyrin IX is not a determinant of plastid-to-nucleus signaling in Arabidopsis. Proc. Natl. Acad. Sci. USA *105*, 15184–15189.

Moulin, M., McCormac, A.C., Terry, M.J., and Smith, A.G. (2008). Tetrapyrrole profiling in Arabidopsis seedlings reveals that retrograde plastid nuclear signaling is not due to Mg-protoporphyrin IX accumulation. Proc. Natl. Acad. Sci. USA *105*, 15178–15183.

Niittylä, T., Messerli, G., Trevisan, M., Chen, J., Smith, A.M., and Zeeman, S.C. (2004). A previously unknown maltose transporter essential for starch degradation in leaves. Science *303*, 87–89.

Ogawa, D., Yamaguchi, K., and Nishiuchi, T. (2007). High-level overexpression of the Arabidopsis *HsfA2* gene confers not only increased themotolerance but also salt/osmotic stress tolerance and enhanced callus growth. J. Exp. Bot. *58*, 3373–3383.

Ohama, N., Kusakabe, K., Mizoi, J., Zhao, H., Kidokoro, S., Koizumi, S., Takahashi, F., Ishida, T., Yanagisawa, S., Shinozaki, K., et al. (2016). The transcriptional cascade in the heat stress response of Arabidopsis is strictly regulated at the level of transcription factor expression. Plant Cell *28*, 181–201.

Pfalz, J., Liebers, M., Hirth, M., Grübler, B., Holtzegel, U., Schröter, Y., Dietzel, L., and Pfannschmidt, T. (2012). Environmental control of plant nuclear gene expression by chloroplast redox signals. Front. Plant Sci. *3*, 257.

Queitsch, C., Hong, S.W., Vierling, E., and Lindquist, S. (2000). Heat shock protein 101 plays a crucial role in thermotolerance in Arabidopsis. Plant Cell *12*, 479–492.

Ragel, P., Streb, S., Feil, R., Sahrawy, M., Annunziata, M.G., Lunn, J.E., Zeeman, S., and Mérida, Á. (2013). Loss of starch granule initiation has a deleterious effect on the growth of Arabidopsis plants due to an accumulation of ADP-glucose. Plant Physiol. *163*, 75–85.

Reiland, S., Finazzi, G., Endler, A., Willig, A., Baerenfaller, K., Grossmann, J., Gerrits, B., Rutishauser, D., Gruissem, W., Rochaix, J.D., and Baginsky, S. (2011). Comparative phosphoproteome profiling reveals a function of the STN8 kinase in fine-tuning of cyclic electron flow (CEF). Proc. Natl. Acad. Sci. USA *108*, 12955–12960.

Roldán, I., Wattebled, F., Mercedes Lucas, M., Delvallé, D., Planchot, V., Jiménez, S., Pérez, R., Ball, S., D'Hulst, C., and Mérida, A. (2007). The phenotype of soluble starch synthase IV defective mutants of Arabidopsis thaliana suggests a novel function of elongation enzymes in the control of starch granule formation. Plant J. *49*, 492–504.

Rott, M., Martins, N.F., Thiele, W., Lein, W., Bock, R., Kramer, D.M., and Schöttler, M.A. (2011). ATP synthase repression in tobacco restricts photosynthetic electron transport, CO2 assimilation, and plant growth by overacidification of the thylakoid lumen. Plant Cell *23*, 304–321.

Schöffl, F., Prändl, R., and Reindl, A. (1998). Regulation of the heat-shock response. Plant Physiol. *117*, 1135–1141.

Sharkey, T.D., and Vanderveer, P.J. (1989). Stromal phosphate concentration is low during feedback limited photosynthesis. Plant Physiol. *91*, 679–684.

Silva-Correia, J., Freitas, S., Tavares, R.M., Lino-Neto, T., and Azevedo, H. (2014). Phenotypic analysis of the Arabidopsis heat stress response during germination and early seedling development. Plant Methods *10*, 7.

Simm, S., Papasotiriou, D.G., Ibrahim, M., Leisegang, M.S., Müller, B., Schorge, T., Karas, M., Mirus, O., Sommer, M.S., and Schleiff, E. (2013). Defining the core proteome of the chloroplast envelope membranes. Front. Plant Sci. *4*, 11.

Sjögren, L.L.E., Tanabe, N., Lymperopoulos, P., Khan, N.Z., Rodermel, S.R., Aronsson, H., and Clarke, A.K. (2014). Quantitative analysis of the chloroplast molecular chaperone ClpC/Hsp93 in Arabidopsis reveals new insights into its localization, interaction with the Clp proteolytic core, and functional importance. J. Biol. Chem. *289*, 11318–11330.

Streb, S., and Zeeman, S.C. (2012). Starch metabolism in Arabidopsis. Arabidopsis Book *10*, e0160.

Streb, S., Egli, B., Eicke, S., and Zeeman, S.C. (2009). The debate on the pathway of starch synthesis: a closer look at low-starch mutants lacking plastidial phosphoglucomutase supports the chloroplast-localized pathway. Plant Physiol. *151*, 1769–1772.

Takizawa, K., Kanazawa, A., and Kramer, D.M. (2008). Depletion of stromal P(i) induces high 'energy-dependent' antenna exciton quenching (q(E)) by decreasing proton conductivity at CF(O)-CF(1) ATP synthase. Plant Cell Environ. *31*, 235–243.

Volkov, R.A., Panchuk, I.I., Mullineaux, P.M., and Schöffl, F. (2006). Heat stress-induced H(2)O (2) is required for effective expression of heat shock genes in Arabidopsis. Plant Mol. Biol. *61*, 733–746.

von Gromoff, E.D., Schroda, M., Oster, U., and Beck, C.F. (2006). Identification of a plastid response element that acts as an enhancer within the Chlamydomonas *HSP70A* promoter. Nucleic Acids Res. *34*, 4767–4779.

Wang, Z.Y., and Tobin, E.M. (1998). Constitutive expression of the *CIRCADIAN CLOCK ASSOCIATED 1* (*CCA1*) gene disrupts circadian rhythms and suppresses its own expression. Cell *93*, 1207–1217.

Cell Reports, Volume *22*

Supplemental Information

Chloroplast Signaling Gates Thermotolerance in *Arabidopsis*

Patrick J. Dickinson, Manoj Kumar, Claudia Martinho, Seong Jeon Yoo, Hui Lan, George Artavanis, Varodom Charoensawan, Mark Aurel Schöttler, Ralph Bock, Katja E. Jaeger, and Philip A. Wigge

Supplementary materials

Supplementary experimental procedures

Plant material and growth conditions

Arabidopsis thaliana WT plants were Columbia-0 (Col-0), Landsberg *erecta* (Ler) or *HSP70-LUC* where specified. The *HSP70-LUC* line has been reported previously (Kumar and Wigge, 2010) and a Ler *pHSP70::LUC* line was generated, by introgression of the *pHSP70::LUC* reporter from Col-0 *pHSP70::LUC.* T-DNA insertion mutants were obtained from the Nottingham Arabidopsis Stock Centre (NASC). Other mutants were have been reported previously: the *CCA1:OX* (Wang and Tobin, 1998) line was obtained from Dr Benoit Landrien (SLCU), *mex1-1* (Niittylä et al., 2004) seeds were obtained from Professor Sam Zeeman (ETH Zurich), and the *ss3/4*, *pgm* and *ss3/4/pgm* (Ragel et al., 2013) lines were obtained from Professor Ángel Mérida (University of Seville).

For general growth, *Arabidopsis* was grown in MTPS walk-in chambers (Conviron) at 22°C long days (16 hr light, 8 hr dark), under 170 µmol m^{-2} s^{-1} Photosynthetically Active Radiation (PAR) and 65% humidity. *Arabidopsis* was grown in Levington F2 compost in either PT24 trays or 7 cm^2 pots. Growth conditions were often specific to the experiment performed and these are described where relevant.

Thermotolerance assays

A thermotolerance assay was developed based on a previously described protocol (Silva-Correia et al., 2014). For each 10 cm^2 plate, 81 seeds were sown with even spacing on 35 ml ½ MS agar and stratified for 2-3 days at 4°C. Seeds were then germinated at 22°C for 24 hrs. After germination, seedlings were grown for seven days in short days (8 hrs light, 16 hrs dark) at 17°C, 22°C or 27°C. After seven days of growth, plates were floated on water in a water bath preheated to 45°C for 30 min. Plates were then put back to the conditions in which the plants were grown before treatment. If the time point when plates were treated was in the dark period, treatment was done with lights off in a dark room; if the heat treatment was done in the light period then treatment was done under ambient laboratory lights (ZT0 treated plants were treated in the light and ZT8 treated plants were treated in the dark (in short days)). Seven days after heat treatment, plants were imaged and the number of plants surviving scored. Survival was defined as the ability to produce new green leaves.

Gene expression analysis

The expression of *HSP70* was assayed by qRT-PCR in multiple experiments. These were broadly split into five categories; timecourse experiments, temperature shift experiments, mutant experiments, detached root and shoot experiments, and drug treatment experiments.

Timecourse experiments were performed at either 17°C, 22°C or 27°C in short days. Seeds were sown on 10 cm^2 plates containing 35 ml of ½ MS (pH 5.7) solid media and stratified for two to three days at 4°C in the dark. Seeds were germinated at 22°C for 24 hrs in short days then transferred to the required growth conditions. The WT short day (Figure 1A) timecourse experiment was grown in a Conviron PGC20 reach-in growth cabinet under 170 µmol m^2 s^{-1} white light. The constant light and dark (Figure 1D), *CCA1:OX* and *cry1/2* experiments (Figure S1C), and chloroplast mutant experiments (Figures 2D and S2E) were grown in a Sanyo-Panasonic MLR-352 growth cabinet under approximately 140 µmol m^2 s^{-1} white light. For timecourse experiments, Zeitgeber time (ZT) 0 indicated lights on and ZT0 samples were sampled in the dark. In short days ZT8 was the end of the day and ZT8 samples were sampled in the light. For constant light and constant dark experiments, plants were entrained to short days for seven days then shifted into constant light or constant dark growth chambers at ZT8 on the seventh day after germination.

For timecourse temperature shift experiments (Figures 1E and 1F) seedlings were grown as for timecourse experiments in Sanyo growth cabinets. On the eighth day after germination at ZT-4, -1, 0, 1, 2, 4, 8, 12 and 16, plates were shifted to 45°C for 30 min in a pre-heated water bath. Shifts at ZT-4, 8, 12 and 16 were performed in the dark, with the water bath in a room with no light, and shifts at 0, 1, 2 and 4 were performed in the light with the water bath under ambient laboratory lights. Samples were also taken just before plants were shifted.

For EMS and T-DNA mutant experiments (Figure 2C), plants were grown as for timecourse experiments in Sanyo cabinets at 17°C. Plates were shifted from a 17°C to 27°C at ZT1 and sampled after one hour at 27°C. A replicate plate was kept at 17°C and sampled at the same time (ZT2).

Root and shoot experiments (Figure S2D) were performed with plants grown vertically for three weeks in short days at 22°C on plate, in Sanyo cabinets. For the shoot and root samples, plants were cut to separate roots from shoots with a razor blade just before sampling.

To test the effects of chemicals on gene expression (Figure 4A), ten seedlings were grown in 12-well tissue culture test plates (Techno Plastic Products) in 500 µl of ½ MS liquid medium (pH 5.7) supplemented with 0.1% w/v glucose. Seedlings were stratified for two to three days at 4°C in the dark, germinated at 22°C for 24 hr and then grown in short days in Sanyo cabinets at either 17°C, 22°C or 27°C. 2-(3,4- dichlorophenyl)-1,1-dimethylurea (DCMU) (Sigma-Aldrich) and 2,5-dibromo-3-methyl-6-isopropyl-p-benzoquinone (DBMIB) (Sigma-Aldrich) were dissolved to 10 mM in 100% ethanol and these 10 mM stocks were diluted to 1 mM in 10% ethanol with sterile H_2O before use. H_2O_2 (Sigma-Aldrich) was diluted to a 50 mM working solution from a 30% w/v (9.8 M) stock in sterile H_2O. On the seventh day after germination plants were treated with 26.25 µl of the chemicals, diluted from 1 mM (DCMU and DBMIB) or 50 mM (H_2O_2) stocks to give the required final concentration in 523.25 µl. Plants were mock treated with either 26.25 µl of 10% ethanol or 26.25 µl of sterile H_2O depending on the chemical used. Plants were treated for the required length of time, depending on the experimental setup, then dried with tissue paper and flash frozen in liquid nitrogen.

For gene expression analysis by qRT-PCR, RNA was extracted from 20-25, seven day old seedlings using a previously described phenol:chloroform extraction method (Box et al., 2011). RNA was treated with RNase free recombinant DNase1 (Roche) to remove contaminating DNA and 1µg of this RNA was used for cDNA synthesis using the Transcriptor First Strand cDNA Synthesis Kit (Roche). Transcript levels were quantified by quantitative Reverse Transcription PCR (qRT-PCR) using SybrGreen (Roche) with 4 µl of cDNA (diluted 10x to a final concentration of 25 ng/µl) as template. Samples were assayed with technical triplicates and were run on a LightCycler 480 (Roche).

For timecourse experiments transcripts of *HSP70* were normalised to the geometric mean expression of *ASCORBATE PEROXIDASE 3 (APX3: AT4G35000)* and *ASPARTIC PROTEINASE A1 (APA1: AT1G11910)* as (Box et al., 2014). For temperature shift experiments transcripts of *HSP70* were normalised to *UBIQUITIN CONJUGATING ENZYME 21 (UBC21: AT5G25760)* (Czechowski et alk., 2005). For experiments where temperature shifts were performed at different times of day, transcripts of interest were normalised against both the geometric mean of *APA1* and *AP3* and against *UBC21*. The data reported are from normalisation against *UBC21* and results were very similar regardless of the reference genes used.

Identifying candidate mutants

A previously described fusion between the promoter of *HSP70 (pHSP70)* and *LUCIFERASE (pHSP70::LUC)* (Kumar and Wigge, 2010) was used to screen for *HSP70* expression in EMS mutagenized *Arabidopsis* Col-0 plants. Luciferase activity was imaged using a Photek HRPCS218 camera and a false colour map of the photon count was overlaid on a bright field image of the screened plate. Seedlings were sprayed with 1 mM D-luciferin free acid (SynChem) with 0.01% Triton X-100, so that all seedlings were covered, and then imaged. Plates were imaged at 17°C at ZT1 and shifted to 27°C for 2 hr and imaged again at ZT3.

Mapping causal genes

Homozygous M3 lines were crossed to Ler *HSP70-LUC* (Line 429) or Ler (line 2641). F2 lines from these crosses were then screened for segregating LUC activity to confirm the recessive nature of these mutations and to generate mapping populations.

Genomic DNA (gDNA) was extracted from a pool of 80-100 plants that showed the mutant phenotype in the F2 mapping populations. For isolation of nuclei and gDNA extraction, approximately 2 g of frozen leaf tissue was ground to a fine powder using pre-chilled pestles and mortars. The powder was re-suspended in 40 ml of ice-cold Honda buffer (25 mM Tris-HCl, pH 7.5, 0.44 M sucrose, 10 mM $MgCl_2$, 0.5% Triton X-100, 10 mM β-mercaptoethanol, 2 mM spermine), vortexed briefly and incubated on ice, with intermittent mixing, for 30 min. The resulting homogenate was filtered through a double layer of Miracloth into a 50 ml Falcon tube. The filtrate was then spun at 2,000 xg at 4°C for 15 min. The resulting pellet was re-suspended in 20 ml of Honda buffer and the suspension spun at 2000 xg for 15 min at 4°C. The resulting pellet was then re-suspended in 20 ml of Honda buffer without spermine and the suspension spun at 2000 xg for 15 min at 4°C. The resulting nuclei pellet was re-suspended in 500 µl of ice-cold TNE (10 mM TrisHCl, pH8.0, 100 mM NaCl, 1 mM EDTA) in four separate tubes. Next, 10 µl of RNaseA (Roche) (20 mg/ml) was added to each 1.5 ml tube (Eppendorf) and

samples were incubated at 65°C for 30 min. Samples were vortexed every 10 min through this heating step. Next 40 µl of proteinase K (AppliChem) (20 mg/ml) was added to each 1.5 ml tube and samples were incubated at 37°C for 60 min; samples were vortexed every 20 min during this heating step. After incubation, 500 µl of phenol:chloroform:isoamyl-alcohol (25:24:1, pH 7.5-8.5) was added and samples were vortexed and incubated on ice for 30 min with further vortexing every 5 min. Samples were then spun at 14,000 xg in a microcentrifuge for 10 min at 4°C. The supernatant was then transferred to new tubes, one volume of isopropanol and 1/10th volume of NaAC (3 M, pH 5.2) were added and samples incubated at -80°C for at least 40 min to precipitate DNA. Samples were then spun at 14,000 xg for 15 min at 4°C and the supernatant discarded. The resulting pellet was washed two times with 500 µl of ice-cold 70% ethanol and samples were spun at 14,000 xg for 5 min at 4°C between washes. The resulting pellet was then dried and re-suspended in 25 µl of TE buffer and the samples from the four separate tubes combined to a final volume of 100 µl. DNA concentration was measured using a Qubit fluorometer (Thermo Fisher scientific) using the high sensitivity, double stranded DNA protocol. DNA quality was assessed by agarose gel electrophoresis and good quality DNA was determined by the presence of a single, bright, high molecular weight band and the absence of a DNA smear.

Libraries for whole genome DNA sequencing were prepared using either the TruSeq DNA low throughput kit (Illumina) or the TruSeq PCR free low throughput kit (Illumina) as the manufacturer's instructions. DNA libraries were sequenced on a NextSeq2000 (Illumina) at the Beijing Genomics Institute (BGI), with 20 to 24 samples pooled per lane and pools sequenced with 100 bp paired end sequencing.

Bioinformatics analysis of the DNA sequencing was performed using CLC genomics (Qiagen). In this description, mapping population refers to pools of plants showing the mutant LUC phenotypes in F2 mapping populations.

Fastq reads from the sequenced mutants and from the parental Col-0 *pHSP70::LUC* line were mapped against the Col-0 (TAIR10) and the Ler reference genomes. Variants were then determined between Col-0 *pHSP70::LUC* and Ler and between the mapping population and Ler using the probabilistic variance detection function. Homozygous SNPs between Col-0 *pHSP70::LUC* and Ler and SNPs of any zygosity between the mapping population and Ler were used for mapping candidate regions. These SNPs were used for mapping candidate regions as they represent differences between the mapping population and Ler that were caused by the differences between the *pHSP70::LUC* parental line and Ler. These SNPs were then filtered for heterozygosity (SNP frequency between 35% and 65%) and for homozygosity (SNP frequency greater than 90%). Frequencies of heterozygous and homozygous SNPs across the genome were then visualised using the CLC visualisation tools, with 100 kb bins of SNP frequency plotted against chromosome position. SNP frequencies were then assessed visually for a region depleted in heterozygous SNPs and enriched for homozygous SNPs against Ler. This region was then classed as a candidate region.

To identify candidate mutations within the candidate region, reads from the mapping population were mapped against the Col-0 TAIR10 genome and variants between the mapping population and TAIR10 were determined using the probabilistic variance detection function. These variants were then filtered for homozygous (SNP frequency greater than 80%, to allow for some mis-scoring of F2 plants) EMS induced (GC to AT) SNPs that caused non-synonymous amino acid changes in the protein they encoded. SNPs that fulfilled these criteria and that were located in the candidate region were classed as candidate mutations.

Confirming causal genes

To identify causal mutations, T-DNA insertion lines in the genes harbouring candidate mutations were genotyped to identify lines homozygous for the required T-DNA insertion. Lines were genotyped using primers designed to bind 100-200 bp upstream (LP) and downstream (RP) of the annotated T-DNA insertion (Table S1). PCR was performed using LP and RP primers and a primer against the left border of the T-DNA insertion (primer LB1.3 (SALK)). Where available, T-DNA lines with insertions in exons of the gene of interest were obtained. These homozygous T-DNA lines were then screened for *HSP70* expression phenotypes by qRT-PCR and T-DNA mutants that had *HSP70* expression phenotypes similar to the corresponding EMS mutant lines were likely to be in the causal genes (As shown for lines 429 and 2641 in Figure 2C).

Transcriptomics

RNA-seq experiments were performed for WT at 17°C, 22°C and 27°C over a 24 h timecourse, *ss4* at 17°C over a 24 h timecourse, *ss3/4, pgm* and *ss3/4/pgm* at 17°C ZT0 and ZT2, and DCMU and DBMIB treated WT at 22°C, ZT1, all in short days. Two independent biological replicates were generated for WT and *ss4* at 17°C,

ss3/4, *pgm* and *ss3/4/pgm* at 17°C and 27°C, and DCMU and DBMIB treatment experiments, there is one biological replicate for WT at 22°C and 27°C. For RNA-seq experiments, all samples were grown in Conviron chambers and sampled as described for *HSP70* expression analysis.

The MagMax RNA extraction kit (Ambion) was used to extract RNA from 20 to 25, eight-day-old seedlings. Extractions were performed as the manufacturer's instructions. RNA was quantified using a Nanodrop 1000-spectrophotometer (Thermo Scientific) and RNA quality was assessed using the 2200 TapeStation system (Agilent) using an RNA1000 screentape (Agilent).

Libraries for RNA sequencing were prepared using either the Illumina TruSeq stranded low or high throughput library preparation kits (Illumina) with 1 µg of RNA as input. Libraries were prepared as the manufacturer's with one minor change; the number of PCR cycles for the DNA enrichment step was reduced from 15 to 13. Library quality was assessed using the Agilent 2200 TapeStation with the D1000 screentape kit.

Sequencing was performed on a NextSeq500 (Illumina) running a final pooled library concentration of 1.8 pM in 1.3 ml. Each pool contained 24 samples and was sequenced using high output, 75 bp, paired end sequencing, generating approximately 1,000,000,000 reads per run.

An in house RNA-seq processing pipeline was used to process the generated sequence data. First, read quality was assessed the quality of reads using FastQC (www.bioinformatics.babraham.ac.uk/projects/fastqc/). Adapter sequences were removed using Trimmomatic (version (v) 3.2) (Bolger et al., 2014), reads mapped to the TAIR10 genome using TopHat (v2.0.10) (Trapnell et al., 2010), duplicate reads removed using Picard's MarkDuplicates (Picard Tools, Broad Institute), transcript abundance estimates, RPKM (Reads Per Kilobase of transcript of transcript per Million mapped reads) and FPKM (Fragments Per Kilobase of transcript per Million mapped reads) were generated using Cufflinks (v2.2.1) (Trapnell et al., 2010) and TPM (Transcript Per Million) values were calculated using the formula described in (Wagner et al., 2012).

TPM values were used for subsequent analysis as they are the least biased estimate of transcript abundance (Wagner et al., 2012). For clustering, the data was filtered to remove both lowly and stably expressed genes using the following parameters. Genes where the sum of the TPMs from all samples in that experiment was less than then number of samples (mean TPM <1) were removed, as were genes where there were a high number of samples with a TPM of 0. The threshold number of 0 was subjective and varied depending on the number of samples in the experiment but roughly if a gene had more than 80% of samples with a TPM of 0 it was removed. A coefficient of variance (CV) (standard deviation of TPM of one gene between samples)/(average of TPM for the same gene between samples) was calculated for each gene and genes with a CV > 0.3 were kept. TPM values were then transformed to enable comparisons between samples by calculating Z-scores ((TPM for one gene in one sample) - (average of TPMs for the same gene between samples))/(standard deviation of TPMs for the same gene between samples) or by calculating Log2 ratios between samples of interest and a control sample (log2 (sample/control)).

The R package DEseq2 (Love et al., 2014) was used to determine differentially expressed genes with the raw read counts from two independent biological replicates used as inputs. For the *ss4* and WT 17°C timecourse, *ss4* samples were used as the samples of interest and WT at the same time points were used as the control samples. Differentially expressed genes were defined as having a log2 ratio of *ss4/*WT more than 0.5 or less than -0.5 and an adjusted p-value less than 0.05. The same cut offs were used for the DCMU and DBMIB experiments, with the mock (ethanol) controls used as the control samples. For the *ss3/4*, *pgm*, *ss3/4/pgm* experiment there were substantially more differentially expressed genes than the other experiments, therefore differentially expressed genes were defined as having a log2 ratio of sample/WT of more than 1 or less than -1, with an adjusted p-value less than 0.05.

Clustering of genes by expression was performed using the 'hclust' function on a 1-Pearson correlation matrix of either Z-scores or log2 ratios (sample/WT) of filtered TPM values and clustering was performed using the complete linkage method. The number of clusters was defined manually and gene expression was visualised using the R function heatmap.2.

Clustering for differentially expressed genes was performed using Z scores for differentially expressed genes. As there were two replicates for the *ss4* and WT at 17°C experiment, Z scores from only one of the replicates were used for clustering.

To determine the enrichments for biological functions in various samples, gene ontology analysis was performed using both the AgriGO tool (Du et al., 2010) and GOrilla (Eden et al., 2009) tools. Genes that passed the filters used when calculating Z-scores for the relevant transcriptomes were used as background.

Motif enrichment analysis was performed using the HOMER2 motif analysis tool (http://homer.salk.edu/homer/) (Heinz et al., 2010). Sequences 1 kb upstream of the transcription start site were analysed using the findmotifs.pl tool to search for enriched known motifs against a background of the 1 kb region upstream of genes that passed the filters used when calculating Z-scores for the relevant transcriptomes

Venn diagrams were produced to assess the numbers of overlapping genes between samples. Venn diagrams were produced using the Venny 2.1 tool (http://bioinfogp.cnb.csic.es/tools/venny/).

ChIP-seq

Plant material was immediately cross-linked when collected using 1% formaldehyde for 15 min under vacuum. Chromatin was extracted from 1 g of cross-linked material. Chromatin was fragmented by sonication using a Bioruptor (Diagenode) in lysis buffer (10 mM Tris-HCl [pH 8], 150 mM NaCl, 1 mM EDTA [pH 8], 0.1% deoxycholate, and 1X protease inhibitor cocktail). ChIP was performed in a buffer containing 20mM Tris-HCl (pH8), 150mM NaCl, 2mM EDTA, 1% triton X-100 and 1X protease inhibitor cocktail using anti-FLAG M2 magnetic beads (Sigma, M8823) coupled to a 1/1 mix of protein-A and protein-G Dynabeads (life technologies, 10001D and 10003D). ChIP-seq libraries were prepared using a TruSeq ChIP Library kit (Illumina) and sequenced on a NextSeq 500 (Illumina).

Chlorophyll fluorescence

The Maxi version of the Imaging-PAM M-series (Walz) was used to determine chlorophyll-a fluorescence parameters of intact plants. The Imaging-PAM was coupled to a custom-made version of the 3010-GWK1 gas exchange chamber (Walz), which can accommodate intact *Arabidopsis* plants in the rosette stage / prior to bolting and allows full control of humidity and temperature. Plants were dark-adapted for 30 min to ensure that all photosystem two (PSII) reaction centres were in the open state. Then, a weak pulse of measuring light (0.1 μmol quanta $m^{-2} s^{-1}$, 1 Hertz frequency) was applied to determine minimal fluorescence (F_o). Plants were then treated with a saturating light pulse (approximately 6000 μmol $m^{-2} s^{-1}$) to put all PSII reaction centres into a closed state and thus to determine maximal fluorescence (F_m). Then, plants were either kept in darkness, or the induction of photosynthesis was followed in actinic light, comparable to the growth light intensity. During photosynthetic induction, steady state fluorescence (F_s) was continuously measured, and maximum fluorescence levels in the light, F_m' , were determined every minute through the course of the experiment. The fraction of open PSII reaction centres according to the lake model of PSII reaction centre connectivity (qL) was calculated according to (Kramer et al., 2004).

Calculating PQ redox state

F_v/F_m and ϕPSII for Col-0, *ss4*, *ss3/4*, *pgm* and *ss3/4/pgm* were reported in (Ragel et al., 2013). The redox state of the PQ pool was calculated from these values using the formula qP= ϕPSII/(F_v/F_m) (Brooks and Niyogi, 2011). The calculated qP values using these measurements were: 0.488 for WT, 0.409 for *ss4*, 0.281 for *ss3/4*, 0.482 for *pgm* and 0.502 for *ss3/4/pgm*.

Supplemental references

Bolger, A.M., Lohse, M., and Usadel, B. (2014). Trimmomatic: A flexible trimmer for Illumina sequence data. Bioinformatics *30*, 2114–2120.

Box, M.S., Coustham, V., Dean, C., and Mylne, J.S. (2011). Protocol: A simple phenol-based method for 96-well extraction of high quality RNA from Arabidopsis. Plant Methods *7*, 7.

Box, M.S., Huang, B.E., Domijan, M., Jaeger, K.E., Khattak, A.K., Yoo, S.J., Sedivy, E.L., Jones, D.M., Hearn, T.J., Webb, A.A.R., *et al.* (2014). ELF3 Controls Thermoresponsive Growth in Arabidopsis. Curr. Biol. *25*, 194–199.

Brooks, M.D., and Niyogi, K.K. (2011). Chloroplast Research in Arabidopsis: Methods and Protocols. Volume II. Methods in Molecular Biology *775*, 299–310.

Czechowski, T., Stitt, M., Altmann, T., and Udvardi, M.K. (2005). Genome-Wide Identification and Testing of Superior Reference Genes for Transcript Normalization. Plant Physiol *139*, 5–17.

Du, Z., Zhou, X., Ling, Y., Zhang, Z., and Su, Z. (2010). agriGO: A GO analysis toolkit for the agricultural community. Nucleic Acids Res. *38*, 64–70.

Eden, E., Navon, R., Steinfeld, I., Lipson, D., and Yakhini, Z. (2009). GOrilla: a tool for discovery and visualization of enriched GO terms in ranked gene lists. BMC Bioinformatics *10*, 48.

Heinz, S., Benner, C., Spann, N., Bertolino, E., Lin, Y.C., Laslo, P., Cheng, J.X., Murre, C., Singh, H., and Glass, C.K. (2010). Simple Combinations of Lineage-Determining Transcription Factors Prime cis-Regulatory Elements Required for Macrophage and B Cell Identities. Mol. Cell *38*, 576–589.

Kramer, D.M., Johnson, G., Kiirats, O., and Edwards, G.E. (2004). New fluorescence parameters for the determination of Q(a) redox state and excitation energy fluxes. Photosynth. Res. 79: 209–218.

Kumar, S.V., and Wigge, P. A (2010). H2A.Z-containing nucleosomes mediate the thermosensory response in Arabidopsis. Cell *140*, 136–47.

Love, M.I., Huber, W., and Anders, S. (2014). Moderated estimation of fold change and dispersion for RNA-seq data with DESeq2. Genome Biol. *15*, 1–34.

Niittylä, T., Messerli, G., Trevisan, M., Chen, J., Smith, A.M., and Zeeman, S.C. (2004). A previously unknown maltose transporter essential for starch degradation in leaves. Science *303*, 87–89.

Ragel, P., Streb, S., Feil, R., Sahrawy, M., Annunziata, M.G., Lunn, J.E., Zeeman, S., and Mérida, Á. (2013). Loss of starch granule initiation has a deleterious effect on the growth of arabidopsis plants due to an accumulation of ADP-glucose. Plant Physiol. *163*, 75–85.

Silva-Correia, J., Freitas, S., Tavares, R.M., Lino-Neto, T., and Azevedo, H. (2014). Phenotypic analysis of the Arabidopsis heat stress response during germination and early seedling development. Plant Methods *10*, 7.

Trapnell, C., Williams, B. a, Pertea, G., Mortazavi, A., Kwan, G., van Baren, M.J., Salzberg, S.L., Wold, B.J., and Pachter, L. (2010). Transcript assembly and quantification by RNA-Seq reveals unannotated transcripts and isoform switching during cell differentiation. Nat. Biotechnol. *28*, 511–5.

Wagner, G.P., Kin, K., and Lynch, V.J. (2012). Measurement of mRNA abundance using RNA-seq data: RPKM measure is inconsistent among samples. Theory Biosci. *131*, 281–285.

Wang, Z.Y., and Tobin, E.M. (1998). Constitutive expression of the *CIRCADIAN CLOCK ASSOCIATED 1 (CCA1)* gene disrupts circadian rhythms and suppresses its own expression. Cell *93*, 1207–1217.

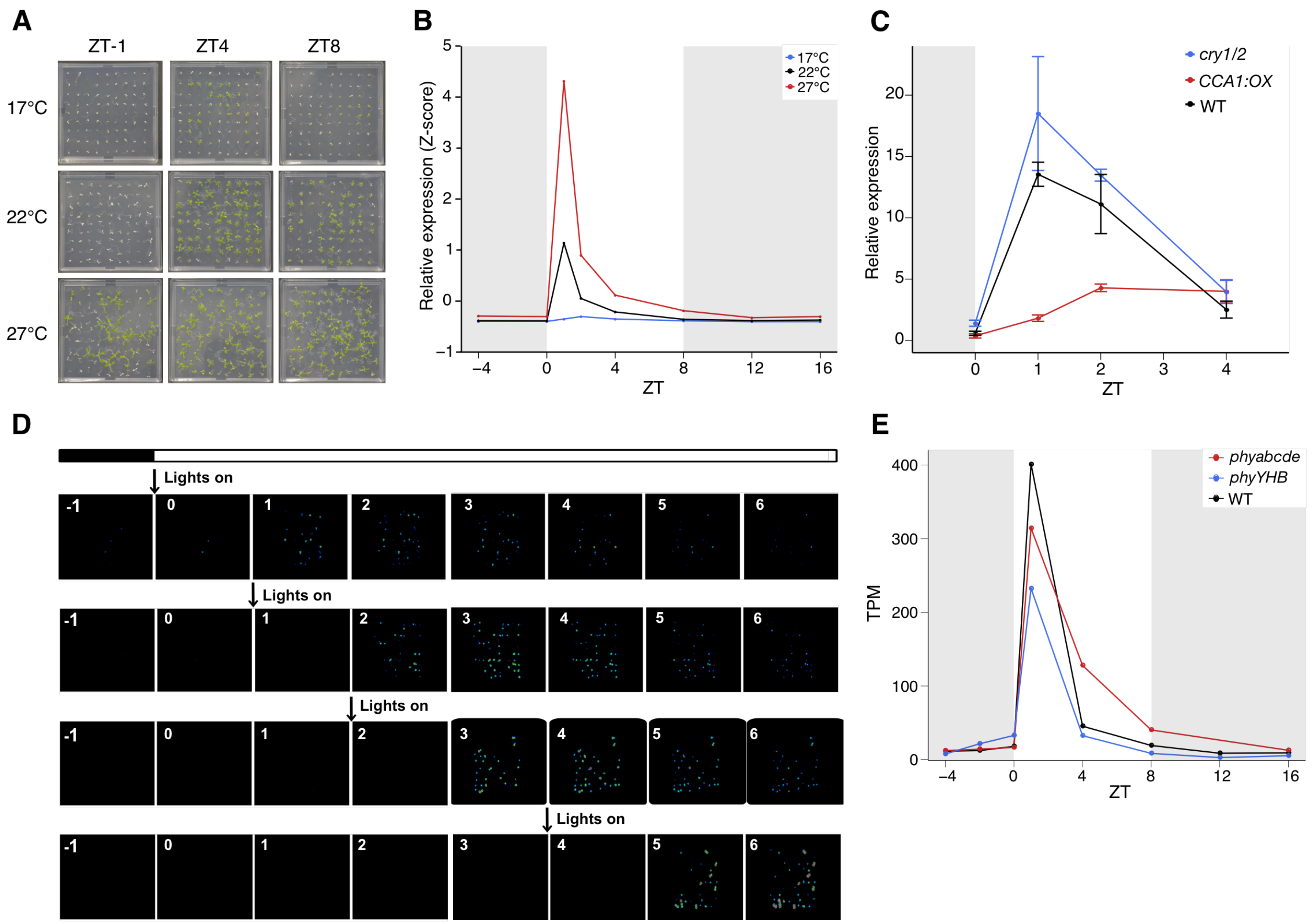
A
ZT-1
ZT4
ZT8
17°C
22°C
27°C
B
Relative expression (Z-score)
ZT
17°C
22°C
27°C
C
Relative expression
ZT
cry1/2
CCA1:OX
WT
D
Lights on
-1
0
1
2
3
4
5
6
E
TPM
ZT
phyabcde
phyYHB
WT

Figure S1 (related to Figure 1)

A) Representative images of WT plants, eight days after heat treatment (45°C for 30 min). B) Expression of *HSP70* in the transcriptomes shown in Figure 1B. Expression shown as Z-scores. WT (Col-0) plants were grown at 17°C, 22°C and 27°C over a 24 h timecourse in short days, grey areas indicated lights off. C) *HSP70* expression assayed by qRT-PCR in WT (Col-0), *cry1/2* and *CCA1:OX* at 22°C ZT0, 1, 2, and 4 in short days. Error bars are + and – SEM (n=3). D) Night extension experiment. LUC activity of *pHSP70::LUC* plants grown in short days at 20°C and imaged at ZT-1, 0, 1, 2, 3, 4, 5, and 6. Each row is a different experiment with 0, 1, 2, and 4 h night extensions into the morning. The black and white bar above the images shows subjective night and day. E) Expression, shown as TPM, of *HSP70* in WT (Ler), *phyabcde* and *phyYHB* plants grown in short days at 22°C. Data from transcriptomes first published in (Jung et al., 2017).

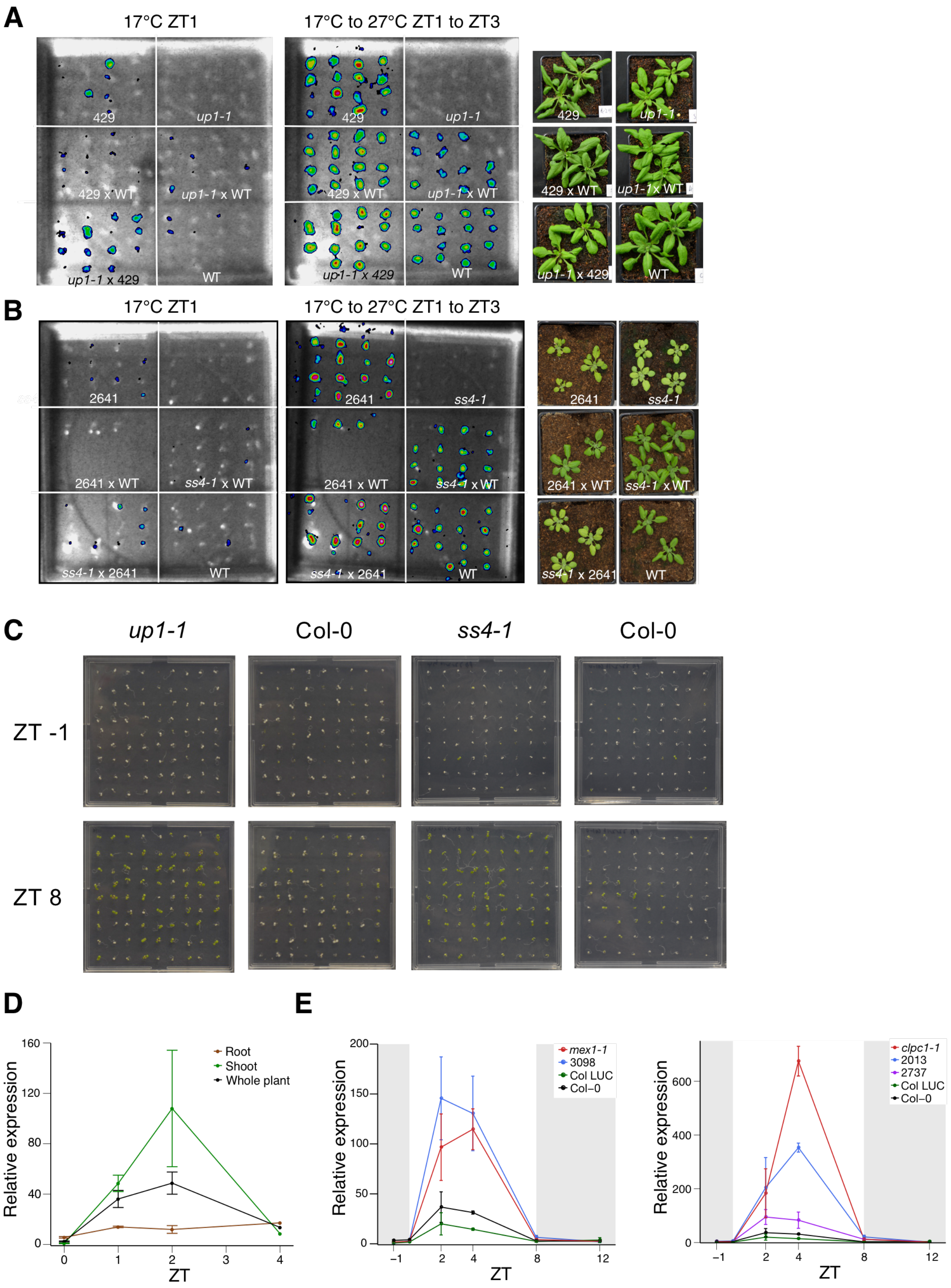
A
17°C ZT1
17°C to 27°C ZT1 to ZT3
429
up1-1
429 x WT
up1-1 x WT
up1-1 x 429
WT
B
17°C ZT1
17°C to 27°C ZT1 to ZT3
2641
ss4-1
2641 x WT
ss4-1 x WT
ss4-1 x 2641
WT
C
up1-1
Col-0
ss4-1
Col-0
ZT -1
ZT 8
D
Relative expression
ZT
Root
Shoot
Whole plant
E
Relative expression
ZT
mex1-1
3098
Col LUC
Col−0
clpc1-1
2013
2737
Col LUC
Col−0

Figure S2 (related to Figure 2)

A) LUC activity of F1 plants from complementation crosses between line 429 and *up1-1* at 17°C ZT1 and after a shift to 27°C for two hours from ZT1 to ZT3. Whole plant phenotypes are shown from the same crosses to the right of the LUC images. WT is Col *pHSP70::LUC.* B) As (A) for line 2641 and *ss4-1*. C) Representative images of *up1-1*, *ss4-1* and WT seedlings after heat treatment started at ZT-1 and ZT8 eight days after heat treatment. D) Expression of *HSP70*, assayed by qRT-PCR, in either roots, shoots, or whole WT plants grown at 22°C in short days and sampled at ZT0, 1, 2, and 4. Error bars are + and – SEM (n=3 for ZT0, 1, 2 and n=1 for ZT4). E) *HSP70* expression assayed by qRT-PCR in *mex1* (line 3098 and *mex1-1*) and *clpc1* (lines 2013 and 2737, and *clpc1*-1) mutants over a timecourse at 17°C in short days sampled at ZT-1, 0, 2, 4, 8 and 12. Grey areas indicate the dark period. Error bars are + and – the range of two measurements (n=2) or SEM (n=3).

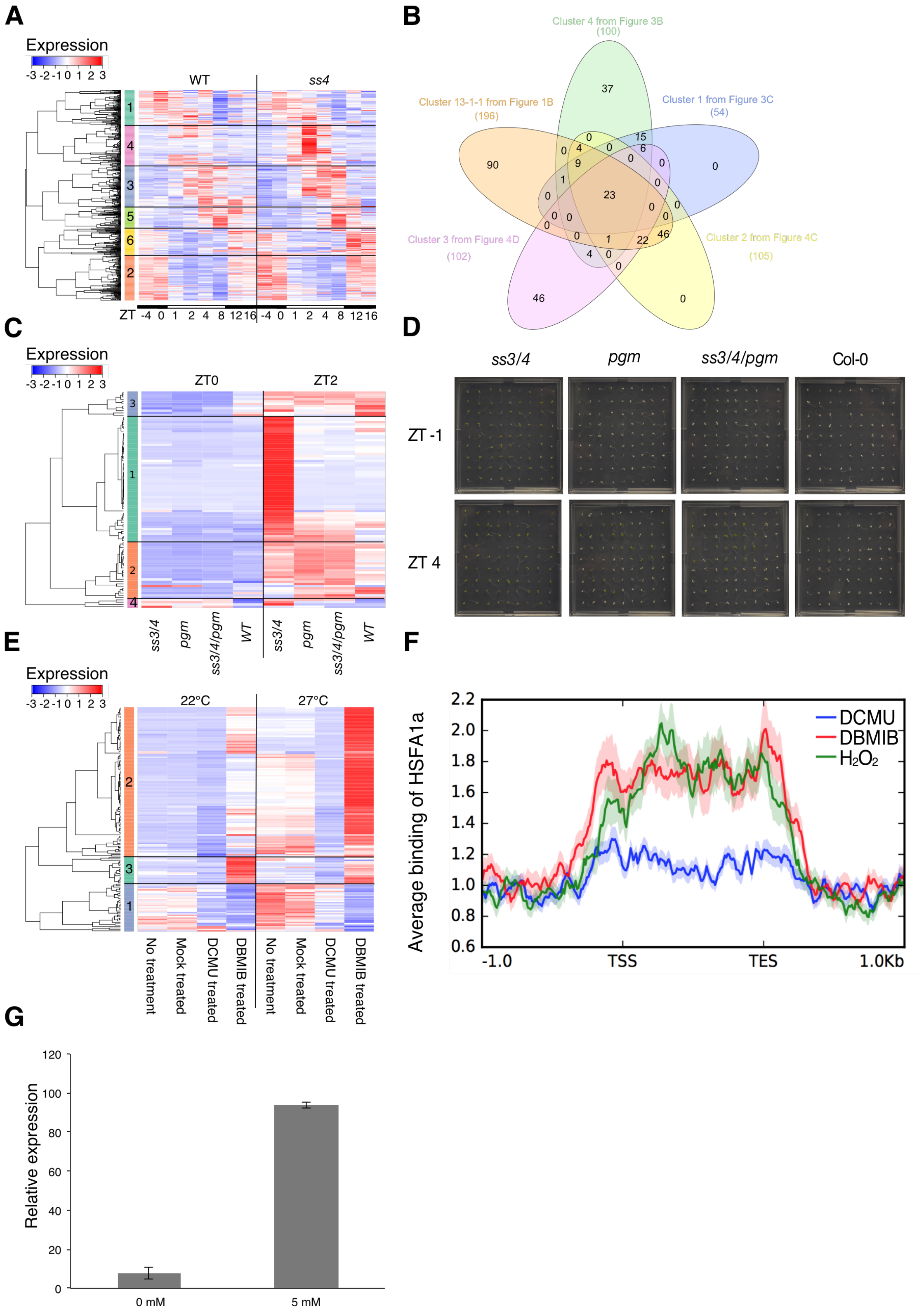
A
Expression
-3 -2 -1 0 1 2 3
WT
ss4
ZT -4 0 1 2 4 8 12 16 -4 0 1 2 4 8 12 16
B
Cluster 4 from Figure 3B (100)
Cluster 13-1-1 from Figure 1B (196)
Cluster 1 from Figure 3C (54)
Cluster 3 from Figure 4D (102)
Cluster 2 from Figure 4C (105)
C
ZT0
ZT2
ss3/4
pgm
ss3/4/pgm
WT
D
ss3/4
pgm
ss3/4/pgm
Col-0
ZT -1
ZT 4
E
22°C
27°C
No treatment
Mock treated
DCMU treated
DBMIB treated
F
Average binding of HSFA1a
DCMU
DBMIB
H2O2
-1.0
TSS
TES
1.0Kb
G
Relative expression
0 mM
5 mM

Figure S3 (related to Figures 3 and 4)

A) Clustering of differentially expressed genes between *ss4* and WT at 17°C over a 24 h timecourse (sampled at ZT-4, 0, 1, 2, 4, 8, 12, and 16) in a replicate of the transcriptomes shown in Figure 3B. Expression is shown as Z-scores and the clusters shown in Figure 3B were used for clustering. B) Overlaps between clusters of genes described throughout the manuscript. C) Clustering of cluster 6 from Figure 3B in a second replicate of transcriptomes of *ss3/4, pgm, ss3/4/pgm* and WT at 17°C ZT0 and 2. First replicate shown in Figure 3C and the clusters from Figure 3C are shown. D) Representative images of *ss3/4*, *pgm*, *ss3/4/pgm* and WT seedlings after heat treatment started at ZT-1 and ZT8 eight days after heat treatment. E) Expression of cluster 13-1-1 from Figure 1B in a second replicate of mock (ethanol), DCMU and DBMIB treated plants at 22°C and 27°C, treated at ZT-1 and sampled at ZT1. Clusters shown are from the first replicate shown in Figure 4C. F) Average binding of HSFA1a to genes with increased expression after DBMIB and decreased expression after DCMU treatments (intersection from Figure 4B) after treatment at ZT-1 with DCMU (30μM), DBMIB (50μM) and H_2O_2 (5mM). Plants were sampled at ZT1. Replicate of the ChIP-seq experiment shown in Figure 4F. G) Expression of *HSP70*, assayed by qRT-PCR, in plants grown at 22°C short days and either mock treated or treated with 5 mM H_2O_2 at ZT-1 and sampled at ZT1. Error bars are the range of two measurements.

Table S1. Genotyping and qRT-PCR primers used in this study (related to Figures 1, 2, 3 and 4)

**Gene**	**T-DNA mutant**	**Genotyping F primer**	**Genotyping R primer2**	**T-DNA primer**
Unknown Protein (UP1) (AT5G08540)	*up1-1* (Salk 100294)	AGGAAAGCTGGAACAGTGCAT	AGCAGATTCTCTCAAGTACAAGT	SALK_LB1.3 - ATTTTGCCGATTTCGG AAC
STARCH SYNTHASE 4 (SS4) (AT4G18240)	*ss4-1* (Gabi-Kat 290D11)	CGGCTCGAAAAGTCTGATGC	ACCATGCAATGCTTCTATTTCGG	GK_LB - CCCATTTGGACGTGAA TGTAGACAC
	ss4-3 (Salk 096130)	CGGCTCGAAAAGTCTGATGC	ACCATGCAATGCTTCTATTTCGG	SALK_LB1.3
ClpC1 (AT5G50920)	*clpc1-1* (Salk 014058)	CGAAACTGGCTGAGGAGGTAG	TAGTTTCAGGGACATCGCCAC	SALK_LB1.3
**Gene**	**qPCR F primer**	**qPCR R primer**		
UBC22 (AT5G25760)	TCCTCTTAACTGCGACTCAGG	GCGAGGCGTGTATACATTTG		
APA1 (AT1G11910)	CTCCAGAAGAGTATGTTCTGAAAG	TCCCAAGATCCAGAGAGGTC		
APX3 (AT4G35000)	GCCGTGAGCTCCGTTCTCT	TCGTGCCATGCCAATCG		
HSP70 (AT3G12580)	CTGACAGCGAGCGTCTCAT	GGATCACTGTATCTTCTTCCGATT		
